# Genome-wide Transcription Factor binding maps reveal cell-specific changes in the regulatory architecture of human HSPC

**DOI:** 10.1182/blood.2023021120

**Published:** 2023-08-18

**Authors:** Shruthi Subramanian, Julie A.I. Thoms, Yizhou Huang, Paola Cornejo, Forrest C. Koch, Sebastien Jacquelin, Sylvie Shen, Emma Song, Swapna Joshi, Chris Brownlee, Petter S. Woll, Diego Chacon Fajardo, Dominik Beck, David J. Curtis, Kenneth Yehson, Vicki Antonenas, Tracey O’Brien, Annette Trickett, Jason A. Powell, Ian D. Lewis, Stuart M. Pitson, Maher K. Gandhi, Steven W. Lane, Fatemeh Vafaee, Emily S. Wong, Berthold Göttgens, Hamid Alinejad Rokny, Jason W.H Wong, John E. Pimanda

**Affiliations:** 1 School of Clinical Medicine, University of New South Wales, Sydney, Australia; 2 School of Biomedical Sciences, University of New South Wales, Sydney, Australia; 3 Centre for Health Technologies and the School of Biomedical Engineering, University of Technology Sydney, Sydney, Australia; 4 Victor Chang Cardiac Research Institute, Sydney, Australia; 5 School of Biotechnology and Biomolecular Sciences, Faculty of Science, University of New South Wales, Sydney, Australia; 6 Macrophage Biology Laboratory, Mater Research, Brisbane, Australia; 7 Bone Marrow Transplant Lab, NSW Health Pathology, Prince of Wales Hospital, Randwick, NSW, Australia; 8 Mark Wainwright Analytical Centre, University of New South Wales, Sydney, Australia; 9 Center for Hematology and Regenerative Medicine, Department of Medicine (MedH), Karolinska Institutet, Huddinge, Sweden; 10 Australian Centre for Blood Diseases, Monash University, Melbourne, VIC, Australia; 11 Blood Transplant and Cell Therapies Laboratory, NSW Health Pathology, Westmead, NSW, Australia; 12 Sydney Children’s Hospital, Sydney, Australia; 13 Centre for Cancer Biology, SA Pathology, University of South Australia, Adelaide, Australia; 14 Adelaide Medical School, University of Adelaide, Adelaide, Australia; 15 Blood Cancer Research Group, Mater Research, University of Queensland, Brisbane, QLD, Australia; 16 Cancer Program, QIMR Berghofer Medical Research, Brisbane, Australia; 17 UNSW Data Science Hub, University of New South Wales, Sydney, Australia; 18 Wellcome - MRC Cambridge Stem Cell Institute, Cambridge, United Kingdom; 19 BioMedical Machine Learning Lab, Graduate School of Biomedical Engineering, University of New South Wales, Sydney, Australia; 20 School of Biomedical Sciences, Li Ka Shing Faculty of Medicine, The University of Hong Kong, Hong Kong Special Administrative Region, China; 21 Haematology Department, Prince of Wales Hospital, Sydney, Australia

**Keywords:** gene regulation, stem cell differentiation, transcriptional networks, haematopoiesis, transcription factors

## Abstract

Hematopoietic stem and progenitor cells (HSPCs) rely on a complex interplay of transcription factors (TFs) to regulate differentiation into mature blood cells. A heptad of TFs − FLI1, ERG, GATA2, RUNX1, TAL1, LYL1, LMO2 − bind regulatory elements in bulk CD34^+^ HSPCs. However, whether specific heptad-TF combinations have distinct roles in regulating hematopoietic differentiation remained unknown. We mapped genome-wide chromatin contacts (HiC, H3K27ac HiChIP), chromatin modifications (H3K4me3, H3K27ac, H3K27me3) and 10 TF binding profiles (the Heptad, PU.1, CTCF, and STAG2) in HSPC subsets (HSC-MPP, CMP, GMP, MEP) and found that TF occupancy and enhancer-promoter interactions varied significantly across cell types and were associated with cell-type-specific gene expression. Distinct regulatory elements were enriched with specific heptad-TF combinations, including stem-cell-specific elements with ERG, and myeloid- and erythroid-specific elements with combinations of FLI1, RUNX1, GATA2, TAL1, LYL1, and LMO2. Furthermore, heptad-occupied regions in HSPCs were subsequently bound by lineage-defining TFs such as PU.1 and GATA1, suggesting that heptad factors may prime regulatory elements for use in mature cell types. We also found that enhancers with cell-type-specific heptad occupancy shared a common grammar with respect to TF binding motifs, suggesting that combinatorial binding of specific TF complexes was at least partially regulated by features encoded in specific DNA sequence motifs. Taken together, this study provides a comprehensive characterisation of the gene regulatory landscape in rare subpopulations of human HSPCs. The accompanying datasets should serve as a valuable resource for understanding adult hematopoiesis and a framework for analysing aberrant regulatory networks in leukemic cells.

## Introduction

Hematopoietic stem cells (HSCs) maintain production of circulating blood cells via their capacity to either self-renew or differentiate to mature cell types ^1^. The most primitive HSCs have multilineage potential but give rise to progenitor cells with increasing lineage restriction. Although single cell analyses have suggested that differentiation occurs over a continuum rather than in discrete leaps ^2–4^, relatively pure populations which correspond to intermediate progenitor stages can be prospectively isolated based on cell surface markers ^4^

Changes in cell identity across differentiation trajectories are directly related to altered transcriptional programs ^5^ which are controlled by lineage-specific gene regulatory networks (GRNs) ^6^. At the simplest level, GRNs are comprised of genes, their associated regulatory elements (promoters and *cis*-regulatory elements (CREs) such as enhancers), and transcriptional regulators, including transcription factors (TF), which bind these elements ^7,8^. Accessibility of regulatory elements is controlled by various chromatin modifications, and the DNA sequence of such elements at least partially determines which TFs can bind ^9,10^. Further control is imposed by chromatin organization into topologically associated domains (TADs) ^11^. Interactions between promoters and their CREs, mediated by chromatin loops and complexes of transcriptional regulators, modulate GRNs and therefore cell identity ^12^.

We previously showed that seven TFs (heptad: FLI1, ERG, GATA2, RUNX1, TAL1, LYL1, and LMO2), all known regulators of healthy and leukemic haematopoiesis ^13–20^, bind combinatorially in bulk CD34^+^ hematopoietic stem and progenitor cells (HSPCs) ^21^ and leukemias ^22–24^. In healthy HSPCs, heptad combinatorial binding occurs at regulatory regions associated with genes involved in stem cell maintenance and function, and also at heptad CREs such that heptad genes form a highly interconnected regulatory circuit ^21,25^. However, study of GRNs in HSPCs is hindered by heterogeneity within the CD34^+^ population and lack of experimental evidence linking promoters to distal regulatory elements.

To address these issues and further our understanding of heptad centred GRNs in blood development, we sorted CD34^+^ HSPCs into HSC/multipotent progenitor (MPP, collectively HSC-MPP), common myeloid progenitor (CMP), granulocyte macrophage progenitor (GMP), and megakaryocyte erythrocyte progenitor (MEP) cell types. We then used chromatin immunoprecipitation followed by sequencing (ChIP-seq) targeting 10 TFs [heptad, PU.1, CTCF, STAG2] and 3 histone modifications [H3K27ac, H3K4me3, H3K27me3], Hi-C, and H3K27ac-HiChIP in each of these cell types to chart the regulatory landscape of human HSPC differentiation. Combinatorial binding of heptad TFs was observed in all sorted populations, although specific patterns of chromatin occupancy differed between cell types. Heptad promoter looping to putative enhancers was variable across cell types, and in many cases combinatorial binding was observed at CREs in immature cells prior to formation of loops in more mature progenitors. Genome-wide occupancy of heptad TFs was also variable across cell types, with distinct sets of CREs enriched for heptad binding in MEP compared to GMP. This variation was at least partially due to sequence motifs in the CREs, with motif composition sufficient to predict cell type with high sensitivity and specificity.

## Methods

Mobilized peripheral blood was collected with patient consent in accordance with the Declaration of Helsinki and used with institutional ethics approval ref:08/190 from the South-Eastern Sydney Local Health District.

Cell sorting, preparation of nuclei, and ChIP were performed essentially as described ^22,26,4^ (detailed in Supplemental Methods). HiC and HiChIP libraries were generated using the Arima Genomics HiC+ kit (Arima cat#A101020).

Bioinformatic analysis used standard pipelines for ChIP ^21^ and HiC/HiChIP data ^27^. Machine learning models were trained using XGBoost ^28^. A UCSC browser session is provided at http://genome.ucsc.edu/s/PimandaLab/Heptad_Regulome. We also provide a tool for data exploration (http://unsw-data-analytics.shinyapps.io/CD34_Heptad_Regulome).

Details of standard experimental and analysis techniques are provided in the Supplemental Methods. Sequencing data have been uploaded to GEO with accession #GSE231486. Contact jpimanda@unsw.edu.au for any original data not included herewith.

## Results

### Genome-wide heptad factor binding in HSPCs

**Figure 1:**
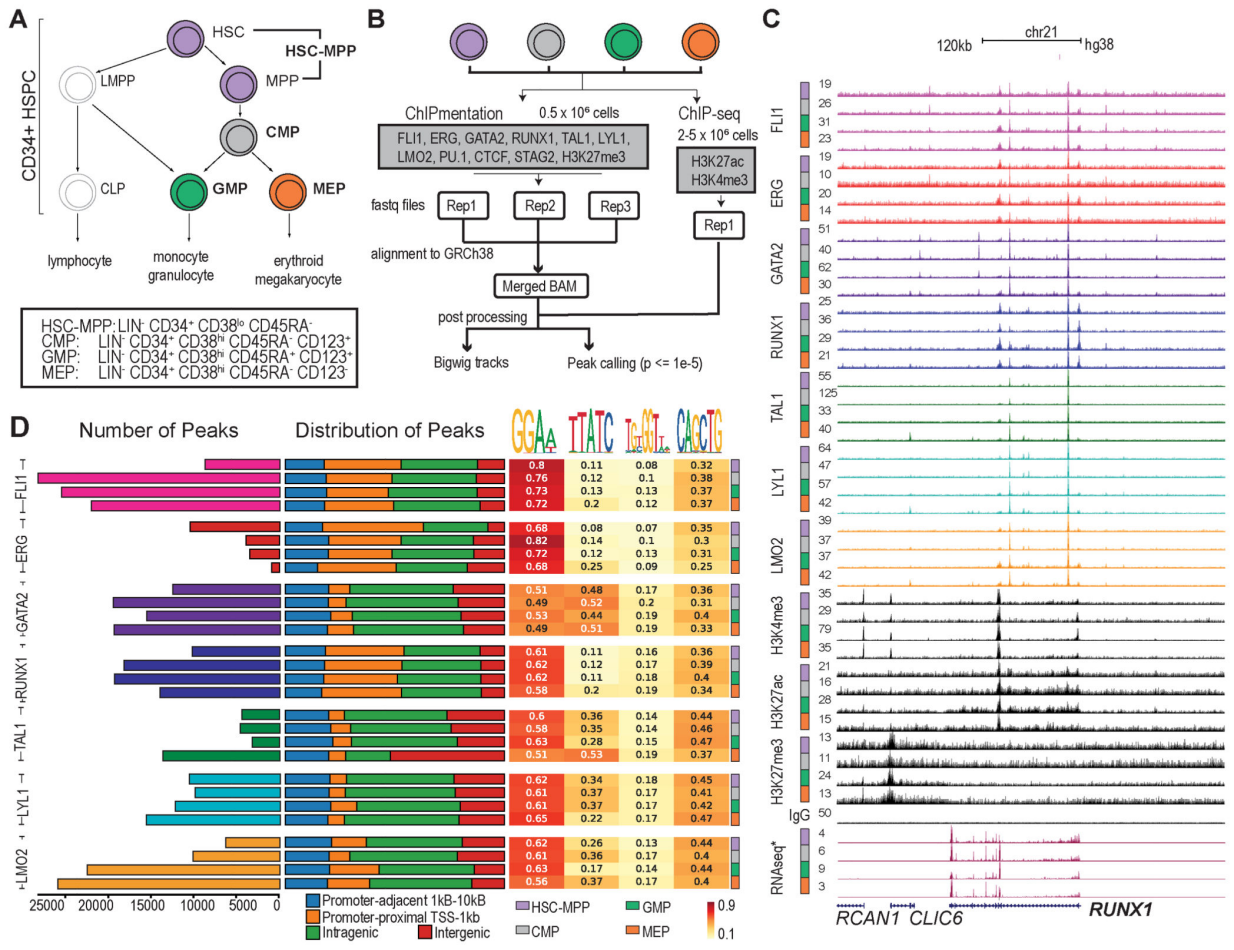
Genome wide patterns of heptad transcription factor binding in fractionated primary human HSPCs. **A)** Human MNCs were isolated from G-CSF-stimulated donors or patients with a non-hematologic malignancy before being enriched for CD34 expression using MACS and further sub-fractionated into individual stem and progenitor cells based on surface marker expression using FACS (colored cells are those studied in this manuscript). **B)** The workflow and analysis pipeline followed for ChIPmentation and ChIP-seq experiments. **C)** UCSC browser track at the *RUNX1* locus (GRCh38 chr21:34,627,969-35,209,177) showing the RPKM-normalized signal from FLI1, ERG, GATA2, RUNX1, TAL1, LYL1, and LMO2, along with H3K4me3, H3K27ac, H3K27me3, IgG (control), and publicly available RNA-seq tracks (GSE75384) for the four cell types. Full UCSC browser tracks are available http://genome.ucsc.edu/s/PimandaLab/HeptadRegulome **D)** Characterization of identified peaks. Number of TF peaks were identified by macs2 (p-value ≤ 1e-5) and their overall distribution along the genome (as percentages of total peaks identified) is shown. Each peak was assigned as either promoter-like (proximal [orange] or adjacent [blue], based on its distance from the TSS), intragenic [green], or intergenic [red], and enrichment (fraction of peaks containing that motif) calculated for the known ETS, GATA, RUNX, and E-Box motifs.

Primary mobilised human CD34^+^ HSPCs were sorted (Figure 1A, Figure S1A), purity checked with colony assays (Figure S1B), and fixed for downstream assays (Figure 1B, Table S1). Cell populations were chosen to span the trajectory from early multipotent stem cells (HSC-MPP) through to progenitors committed to the myeloid (GMP) or erythroid (MEP) lineages.

High quality ChIP data were obtained for all heptad factors (Figure 1C, Figure 1D, Table S2, http://genome.ucsc.edu/s/PimandaLab/Heptad_Regulome). Total peak numbers were highly variable between heptad factors and across cell types (Figure 1D). We observed cell-type-specific trends consistent with known expression patterns and biology. For example, ERG peaks were most abundant in HSC-MPPs, consistent with its role in maintaining the stem cell state ^29,30^, while TAL1 peaks were most abundant in MEP, consistent with its role in erythroid development (Figure 1D). Distributions of TFs across genome features were generally conserved across cell types, but differed between heptad factors. For example, FLI1, ERG, and RUNX1 peaks were located at both promoter and non-promoter regions (Figure 1D), while GATA2, TAL1, LYL1, and LMO2 peaks were predominantly located at non-promoter regions. TAL1 peak distribution in MEP was unique, with many peaks found at intergenic regions. Motif enrichment analysis showed ETS (GGAA) and E-Box (CANNTG) motifs in TF-occupied regions from all factors. FLI1, ERG, and RUNX1 peaks were highly enriched for the ETS motif, while GATA2, TAL1, LYL1, and LMO2 peaks showed additional enrichment for the GATA motif (GATA), particularly for GATA2 and TAL1 in MEP. Consistent with observations in bulks HSPCs^21^ enrichment of RUNX1 motifs was minimal. Overall, we observed conserved patterns of heptad binding, but also distinct differences between factors and across cell types, consistent with dynamic remodeling of the heptad network across the HSPC differentiation trajectory.

### Combinatorial binding of heptad TFs is cell-type-specific

**Figure 2:**
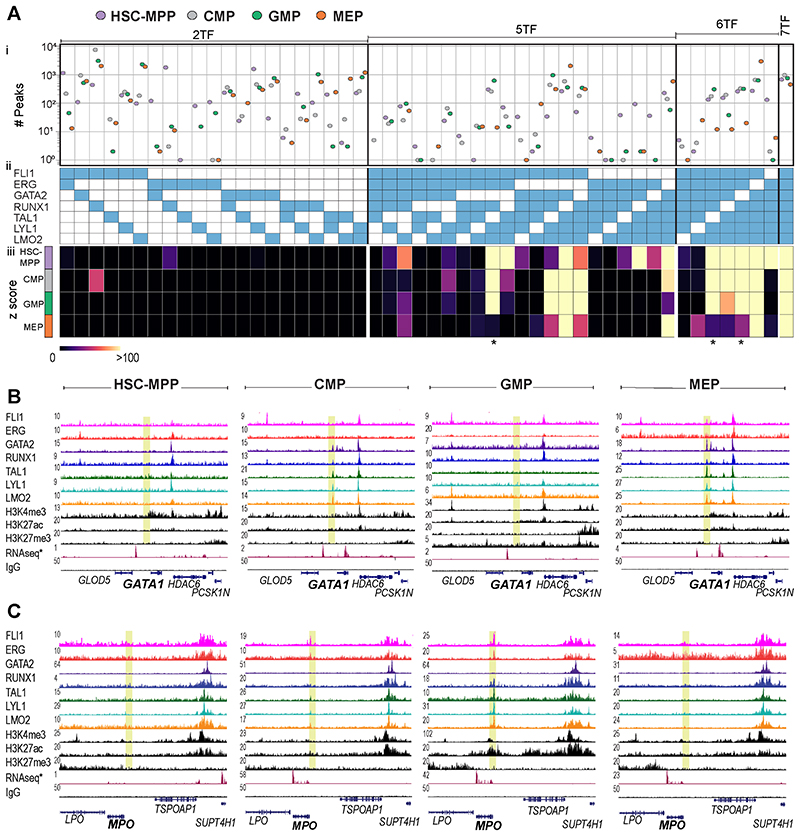
Combinatorial binding of heptad transcription factors is cell-type specific. **A)** A composite graph with three components: i) number of combinatorial binding peaks identified in the four cell types, for ii) combinations of 2, 5, 6, and 7 heptad factors and iii) heatmap showing z-scores for the combinations presented in (ii). Star indicates combinations lacking GATA2 and/or TAL1. **B-C)** UCSC browser tracks showing RPKM-normalized signal tracks of the heptad factors, H3K4me3, H3K27ac, H3K27me3, RNA-seq (public data: GSE75384), and IgG (control) in HSC-MPP, CMP, GMP, and MEP (left to right), at **B)** the *GATA1* locus (GRCh38 chrX:48,724,037-48,839,866), a gene vital for erythroid lineage specification, and at **C)** the *MPO* locus (GRCh38 chr17:58,238,087-58,348,896), a gene specific to the monocytic lineage.

We previously described combinatorial binding of heptad factors in bulk human HSPCs ^21^, and now extend these observations to specific cell populations. We quantified ChIP peaks containing all possible combinations of two or more heptad factors (Figure 2Ai, ii), and evaluated the probability these occurred by random chance in each cell type (Figure 2Aiii). As in bulk HSPCs ^21^, combinatorial binding of all seven factors was the most significant event in any of the four cell types (z-scores for all seven factors; HSC-MPP: 8199.94, CMP:6314.92, GMP:3543.93, MEP:1877.48). Pairwise combinations had low significance scores, with exceptions such as ERG/RUNX in HSC-MPPs. Higher order complexes generally had higher significance scores, with 5/7 possible six-factor complexes showing highly significant binding in at least one cell type. Specific combinations broadly matching known TF function were also observed. For example, five- or six-factor combinations lacking GATA2, TAL1, or both, had low significance scores in MEPs (Figure 2A, stars). We also asked whether PU.1 showed combinatorial binding with heptad factors. As previously observed ^25^, co-binding of single heptad factors with PU.1 was minimal (Figure S2A), while addition of PU.1 to 6/7 TF combinations modestly accentuated existing patterns (Figure S2B). Overall, we find that combinatorial binding of heptad TFs is a general feature of stem and progenitor cells, with some combinations restricted to specific cell types.

Given the cell-type-specificity of some TF combinations we predicted that dynamic formation of TF complexes might play a role in priming CREs for subsequent activation. We explored heptad factor binding at promoter regions of two lineage-specific genes: the erythroid regulator *GATA1* and the monocyte gene *MPO*, neither of which showed heptad binding in HSC-MPPs. At the *GATA1* promoter (Figure 2B; yellow region), GATA2, RUNX1, TAL1, LYL1, and LMO2 binding was observed in CMP. As cells transitioned to MEP, binding peaks became more prominent and now included FLI1 and ERG. However, there was essentially no heptad binding at the *GATA1* promoter in GMP. At the *MPO* promoter (Figure 2C; yellow region), FLI1, ERG, TAL1, LYL1, and LMO2 binding was observed in CMP. Binding peaks became more prominent as cells transitioned to GMP, with RUNX1 also bound, but no TF binding was observed at the *MPO* promoter in MEP. Taken together our data suggests that distinct patterns of heptad TF binding may prime the genomic landscape of human blood stem cells towards either an erythroid or myeloid fate.

### Heptad regulatory circuits are remodelled during myeloid progenitor development

Genes encoding heptad TFs form a densely interconnected regulatory circuit in bulk HSPCs ^21^, and chromatin accessibility at heptad gene CREs is sufficient to predict blood cell identity ^23^. To better understand genome organization and connectivity at heptad loci during HSPC development we performed HiC and H3K27ac HiChIP experiments on HSC-MPP, CMP, GMP, MEP. While the majority of genome compartments remained stable across all cell types, some compartments underwent B to A, or A to B switching upon transition from CMP to lineage-committed progenitor (Figure S3Ai). Notably, some regions underwent compartment switching only in GMP, with corresponding changes in H3K27ac signal (Figure S3Aii, iii). TAD boundaries were highly conserved between cell types (Figure S3B), and HiC contact matrices around heptad gene loci were also highly similar (Figure S4). Global CTCF and STAG2 binding was also conserved across cell types (Figure S5). Overall, consistent with previous reports ^31^ we observed minimal variation in high level genome organization between HSPC subpopulations.

H3K27ac HiChIP experiments generated thousands of significant interactions with FDR ≤ 0.01 (HSC-MPP: 26210, CMP: 8170, GMP: 43448, MEP: 32773; Table S3). We focused on loops where at least one interacting region was annotated as a promoter (P). As this experiment enriched fragments with the H3K27ac active enhancer mark, we consider looped CREs as putative enhancers (E). We filtered promoter-enhancer (P − E) loops based on presence of an ATAC peak at the distal enhancer and integrated these with ChIP-seq data to create regulatory network maps at each heptad gene locus for each cell type.

**Figure 3:**
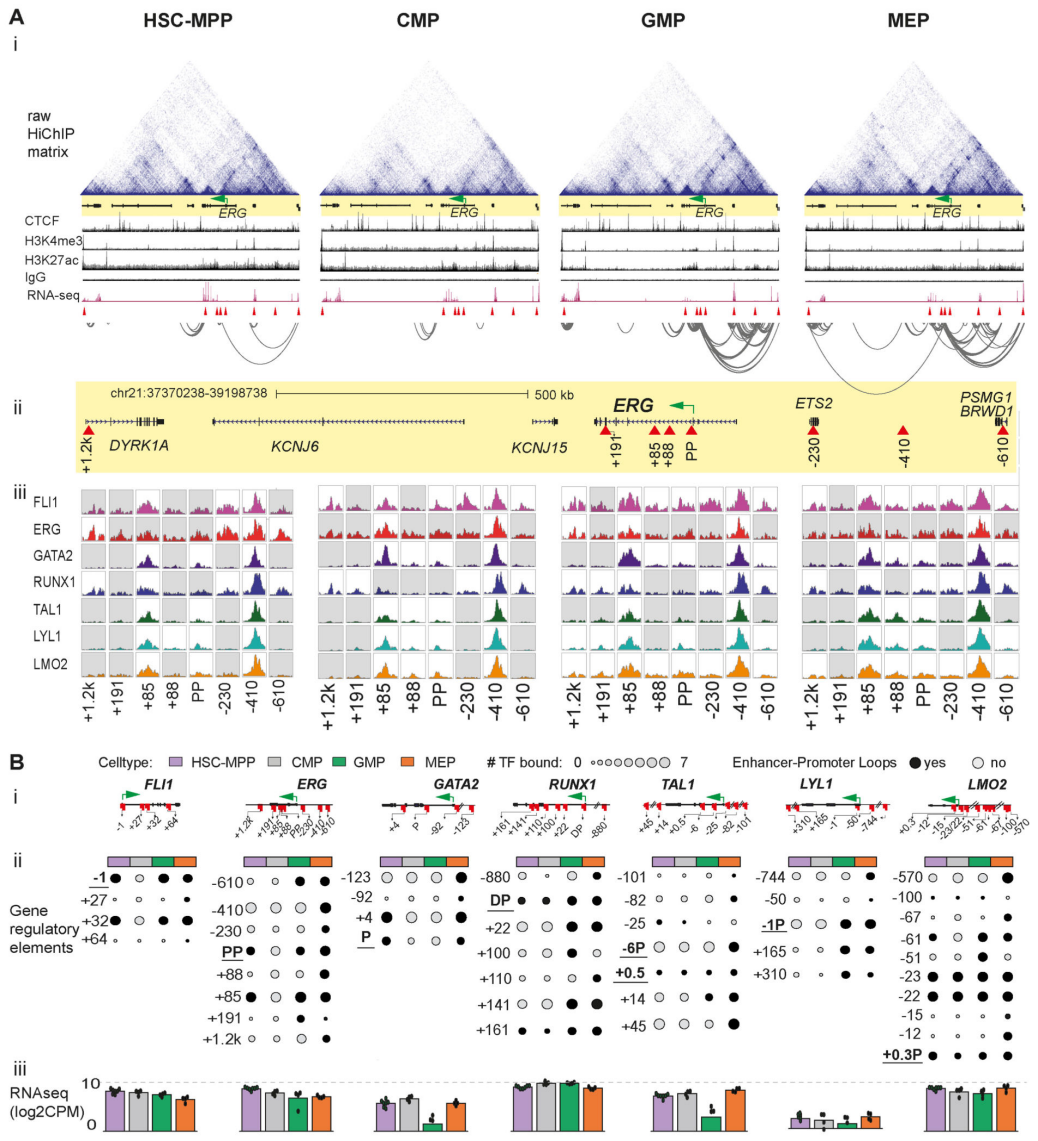
Heptad regulatory circuits are remodelled during myeloid progenitor development. **A)** Step-wise identification of potential regulatory regions interacting with the ERG promoter: i) Raw HiChIP contact matrix, CTCF, H3K4me3, H3K27ac, IgG, RNA-seq, and significant H3K27ac HiChIP interactions (FDR ≤ 0.01) at the *ERG* locus (GRCh38 chr21:37370238-39198738). The *ERG* promoter is indicated by the green arrow (only those HiChIP interactions where both interacting ends were found at the given locus are shown). ii) Magnified view of the *ERG* locus, with regulators identified to loop to the *ERG* proximal promoter shown as red triangles. iii) FLI1, ERG, GATA2, RUNX1, TAL1, LYL1, and LMO2 peaks at the defined regulators in each individual cell type. The peaks shown are RPKM-normalised and white boxes indicate presence of a computationally called ChIP-seq peak at the specific region. **B)** Summary plot of gene regulatory interactions across the heptad genes: i) Individual heptad gene loci with identified regulators indicated by red markers. ii) Dot plots showing regulatory regions as rows and the four cell types as columns, with size of the dot indicating number of heptad factors bound and bold color indicating the presence of an active regulatory link to the promoter (using H3K27ac HiChIP). Promoters are underlined. iii) Bar plots with individual replicates showing average log2 counts of relevant heptad gene expression in the four cell types (GSE75384).

Promoter-enhancer loops corresponding to the heptad spanned wide genomic regions; around 500kb for *FLI1, GATA2*, and *TAL1* and 1-2Mb for *ERG, RUNX1, LYL1*, and *LMO2* (Figure 3Ai, ii, Figure S6). Overall, we detected multiple putative enhancers for the heptad genes *ERG* (−610/−410/−230/+85/+88/+191/+1200), *FLI1* (+27/+32/+64), *GATA2* (−123/−92/+4), *RUNX1* (−880/+22/+100/+110/+141/+161), *TAL1* (−101/−82/−25/+0.5/+14/+45), *LYL1* (−744/−50/+165/+310), and *LMO2* (−570/−100/−67/−61/−51/−23/−22/−15/−12) (Figure 3Ai, ii, Figure S6, Table S4. Some regions have been described in humans and/or in mice^32,33,17,21,34–45^ while others were novel (Table S4). Two known heptad enhancers were not directly looped to their corresponding promoter (*FLI1*-15, *GATA2-117*, Figure S6Ai, S6Bi) ^21,44^, although we did observe looping between *GATA2*-117 and other putative enhancers in MEPs (Figure S6Bi). Furthermore, looping at heptad genes generally increased in complexity in GMP and MEP compared to HSC-MPP, with the sparsest looping observed in CMP (Figure 3Ai, Figure S6). This additional complexity often included extensive looping to CREs not directly connected to promoters, suggesting that heptad gene expression is fine-tuned by highly interconnected cell-specific enhancer communities (e.g., TAL1 locus in MEP (Figure S6Di), LYL1 locus in GMP and MEP (Figure S6Ei)).

Most directly looped elements showed heptad factor binding in at least one cell type (Figure 3Aiii, Figure S6). Some core CREs showed extensive heptad binding in all cell types, including known and functionally validated enhancers such as *ERG+85, GATA2+4, RUNX1+22*, and *TAL* +45 (Figure 3Aiii, Figure S6Biii, Ciii, Diii, Table S4), plus novel CREs such as *ERG*-410 and *LMO2*-570 that can now be linked to heptad genes (Figure 3Aiii, Figure S6Fiii). Integration of HiChIP and ChIP data at heptad gene loci showed diverse patterns of looping and TF binding across the four cell types (Figure 3Bi, ii). At the *ERG* locus, the *ERG+*85 enhancer was linked to the proximal promoter in HSC-MPP, GMP, and MEP, while other *ERG* elements showed promoter looping in GMP and/or MEP. (Figure 3Bii). Furthermore, heptad TF binding often occurred in HSC-MPPs with subsequent promoter looping of that element in more differentiated cell types (e.g./ *GATA2−123, TAL1+45*, and *LMO2*-570, Figure 3Bii). However, these epigenetic changes did not directly correspond to the steady state transcriptional output of heptad genes, which was relatively stable across the four cell types (Figure 3Biii).

Analysis of bulk HSPCs found that heptad genes regulate themselves and each other via a densely interconnected auto-regulatory circuit ^21^. We used our expanded set of heptad CREs to construct network connectivity maps for each cell (Figure S7). These maps show that although the transcriptional output of the heptad genes was relatively stable across HSC-MPP, CMP, GMP, MEP, there were cell-type specific differences in *cis*- and *trans*-regulatory mechanisms by which stable expression was maintained. Overall, our data set allowed us to observe extensive remodeling of the regulatory connections within and between individual heptad genes during hematopoiesis and increases our understanding of the complex network regulating heptad genes during hematopoiesis.

### Heptad TFs and regulation of lineage-specific gene expression

**Figure 4:**
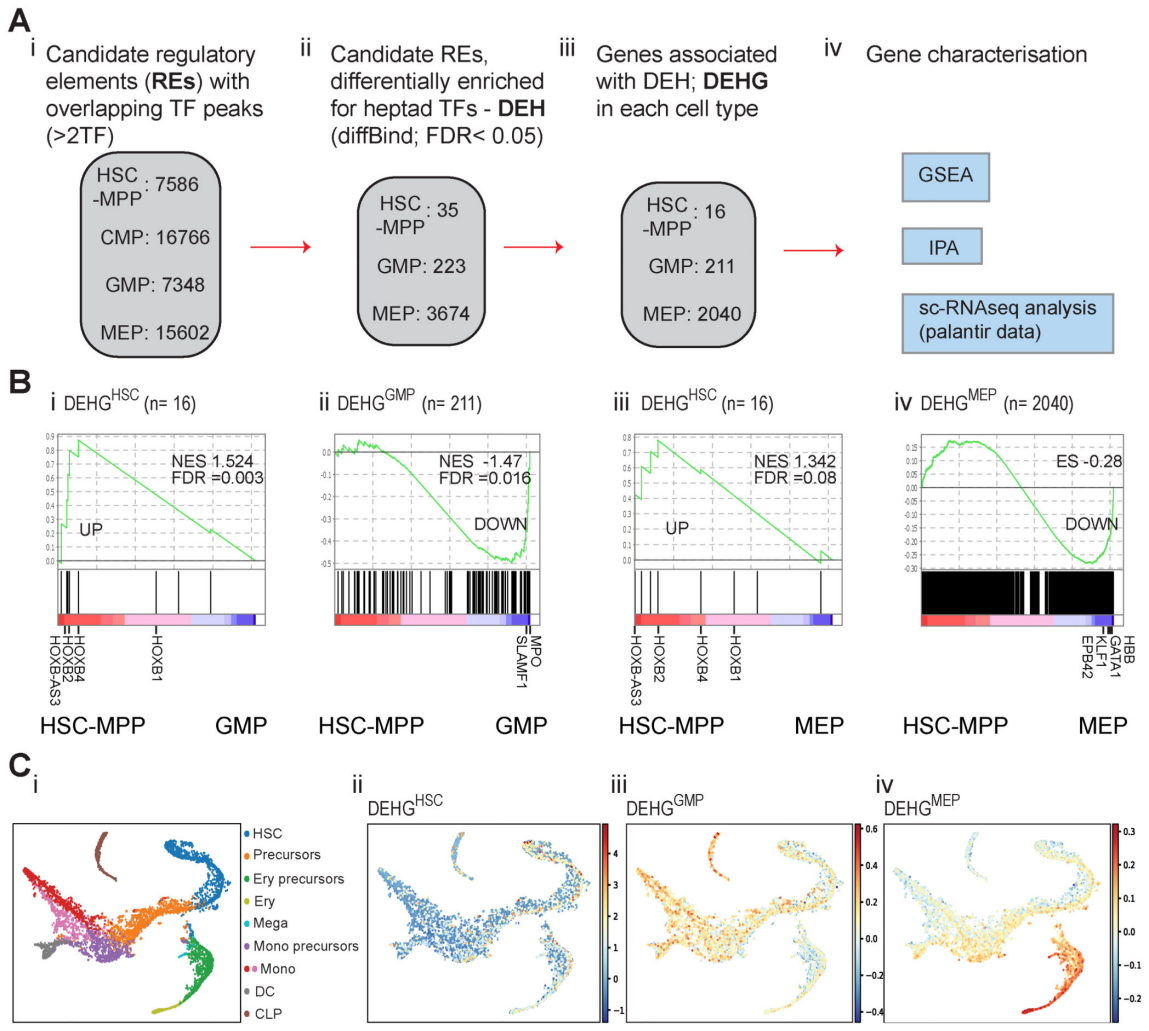
The role of heptad transcription factors in regulating lineage-specific gene expression. **A)** Schematic of the bioinformatic strategy used to derive regions showing differential heptad factor binding: i) Candidate regulatory elements (REs) with binding of at least two heptad factors were chosen in the four cell types; and ii) DiffBind was used to filter for regions showing differential enrichment for heptad factors with an FDR < 0.05. To perform DiffBind analysis only HSC-MPP (HSC), GMP, and MEP populations were chosen. iii) These DEH (differentially enriched for heptad) regions were linked to genes either directly (present across a 10 kb promoter region) or indirectly (distal links using significant [FDR < 0.01] H3K27ac- HiChIP interactions), and iv) used as input for multiple characterisation assays. **B)** GSEA plots showing enrichment of derived gene sets in pairwise gene expression comparisons: i) DEHG^HSC^ (genes linked to DEH regions in HSC-MPP) enriched in HSC-MPP with respect to GMP, ii) DEHG^GMP^ enriched in GMP with respect to HSC-MPP, iii) DEHG^HSC^ enriched in HSC-MPP with respect to MEP, and iv) DEHG^MEP^ enriched in MEP with respect to HSC-MPP. NES: normalized enrichment score, ES: enrichment score, q = FDR q value from GSEA. **C)** Scoring cell-specific DEHGs along a i) single-cell expression map reveals localized enrichment of expression: ii) DEHG^HSC^, iii) DEHG^GMP^, and iv) DEHG^MEP^.

We next asked whether heptad factor chromatin occupancy was associated with cell-type-specific transcriptional output. We identified genomic regions with cell-type-enriched binding of at least 2 heptad TFs (Differentially Enriched for Heptad [DEH]) and linked these to genes using HiChIP data (Genes associated with DEH [DEHG]) (Figure 4A, Table S5A). Approximately half of the regions were promoter-like (up to 10 kb upstream of a TSS). To characterize candidate regulatory elements (REs) and their associated genes we conducted gene set enrichment analysis (GSEA), ingenuity pathway analysis (IPA), and single-cell analysis. DEHG in HSC-MPP/GMP/MEP had greater expression in their respective cell types compared to other cell types (Figure 4B) and included previously reported lineage specific genes such as *SLAMF1* and *MPO* in GMPs ^46,47^ and *GATA1* and *KLF1* in MEPs^48–50^. Furthermore, genes differentially bound in GMP were enriched for pathways linked to myelopoiesis and granulopoiesis (Figure S8A) while genes linked to differentially bound regions in MEP were enriched for pathways linked to erythropoiesis (Figure S8B). Only 16 HSC-MPP-specific genes were identified precluding pathway analysis. However, these genes included stem cell regulators ^51^ such as *HOXB1, HOXB2, HOXB4* (Table S5A).

We also explored expression of DEHG in single cells across hematopoietic differentiation (Figure 4Ci) ^3^. HSC-MPP-associated DEHG (DEHG^HSC^) were enriched in cells annotated as HSC clusters (Figure 4Cii), while DEHG^GMP^ were enriched in monocyte clusters (Figure 4Ciii) and DEHG^MEP^ in erythroid lineage cells (Figure 4Civ). Together these data support our hypothesis that heptad occupancy at regulatory elements can be linked to cell-specific transcriptional output.

### Heptad TF binding at regulators of genes crucial for myeloid and erythroid cell development

**Figure 5:**
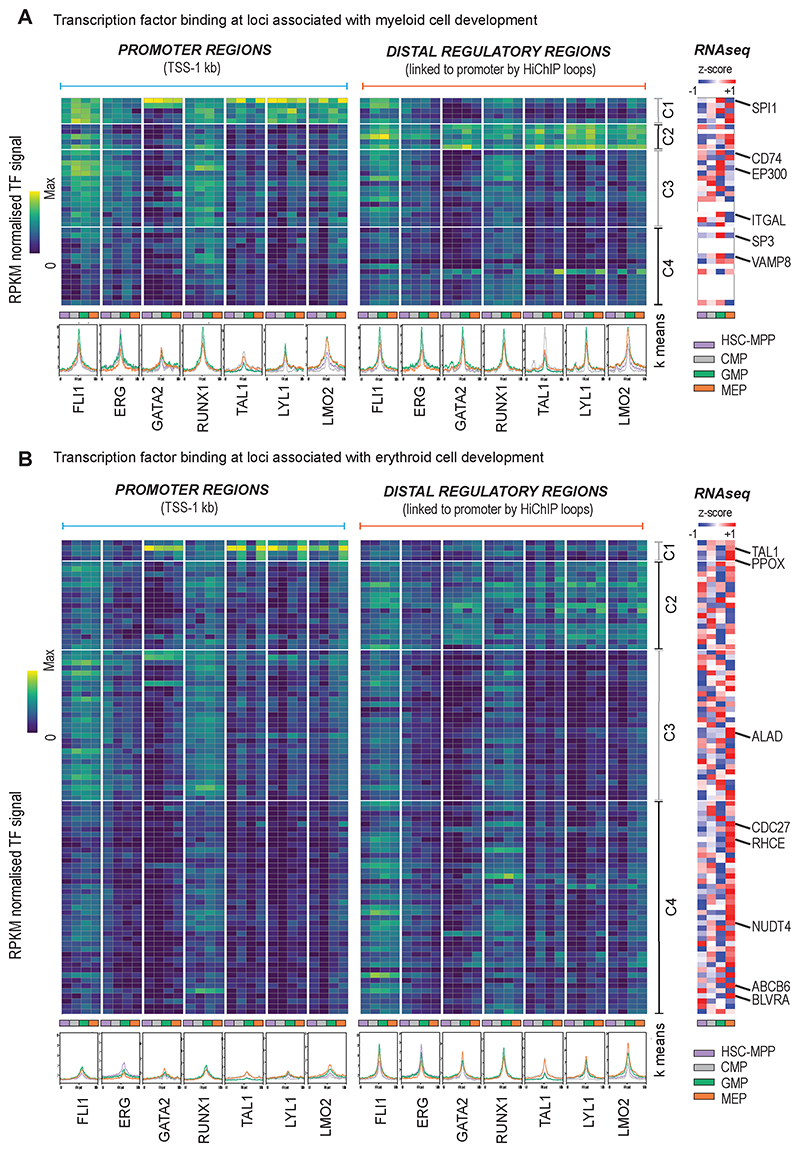
Heptad transcription factors at promoters and distal regulators of genes crucial for myeloid and erythroid cell development. **A)** Genes associated with myeloid development. *Left*: *k*-means clustered heatmaps of TF binding intensity at promoters and distal regulatory regions. Profile plots show normalised signal for each TF in each cell type at the regions depicted in the heatmap. *Right*: z-score normalised heatmaps of RNA-seq counts (GSE75384) for the corresponding gene in each cell type. White rows are genes with no expression values in the dataset. **B)** Genes associated with erythroid development. *Left*: *k*-means clustered heatmaps of TF binding intensity at promoters and distal regulatory regions. Profile plots show normalised signal for each TF in each cell type at the regions depicted in the heatmap. *Right*: z-score normalised heatmaps of RNA-seq counts (GSE75384) for the corresponding gene in each cell type.

We next asked whether we could detect specific patterns of heptad occupancy at known lineage-specific genes with roles in mature monocyte and granulocyte maintenance (myeloid cell development), linked to erythroid cell development and heme metabolism (erythroid cell development), and linked to stem cell function (Table S5B). From these lists we focused on genes whose promoters were looped to a putative enhancer in any of our HiChIP datasets. We identified 40 P-E pairs from the myeloid genes, 91 from the erythroid genes, and 81 from the stem cell genes (Table S5B) and used *k*-means clustering of TF binding signals to compare heptad occupancy patterns at each P-E pair in each cell type. We observed variable TF binding across associated promoter and enhancer regions. Genes in cluster 1 (C1) showed TF enrichment at promoters, genes in cluster 2 (C2) showed TF enrichment at enhancers, and genes in clusters 3 and 4 (C3, C4) had TF enrichment at both promoters and enhancers (Figure 5A, Figure 5B, Figure S9A). Furthermore, several TF-specific observations were evident. First, ERG occupancy at stem cell, myeloid, and erythroid genes was generally highest in HSC-MPPs, and to a lesser extent GMPs, consistent with its role in maintaining stem cells (Figure S9B, Table S6) ^30,23,29^ Second, FLI1 and RUNX1 were bound across both myeloid- and erythroid- gene associated regions across all differentiation stages (Figure S9C). Third, GATA2, LYL1, and LMO2 have increased occupancy at myeloid- and erythroid-specific regulatory regions during lineage commitment (Figure S9D, Table S6). Fourth, high TAL1 occupancy was observed in MEPs and their precursor CMPs, and this occurred at regions linked to erythroid genes and regions linked to myeloid genes (Figure S9E, Table S6). Binding in CMPs may indicate a role for TAL1 in priming regulatory regions and recruiting activators or repressors in downstream cell types. Finally, there was a general pattern of heptad TF occupancy at promoters and enhancers of lineage-specific genes even in the earliest stem cells, suggesting that heptad factors bind lineage-specific regulatory regions prior to lineage commitment and subsequent differentiation.

To further explore whether heptad binding in differentiated cell types is initiated in early stem cells, we identified ChIPseq peaks bound by a single heptad factor in HSC-MPPs and compared TF binding density at these regions across cell types. Regions with peaks in HSC-MPPs had additional heptad factors bound in committed progenitors. For example, peaks bound by ERG in HSC-MPPs (Figure S10A) showed prominent binding of FLI1 and RUNX1, in GMPs, and binding of all heptad factors except for ERG in MEPs (Figure S10B, C). Furthermore, regions bound by single heptad TFs in HSC-MPPs were subsequently bound by PU.1 in dendritic cells or GATA1 in proerythroblasts (Figure S10D). Overall, these data support dynamic assembly of heptad factor complexes at sites subsequently occupied by lineage-determining TFs. Further experimentation will resolve whether heptad factors actively prime CREs for utility in more mature cell types.

### Regulatory regions with cell-type-specific heptad occupancy have distinct epigenetic features

**Figure 6:**
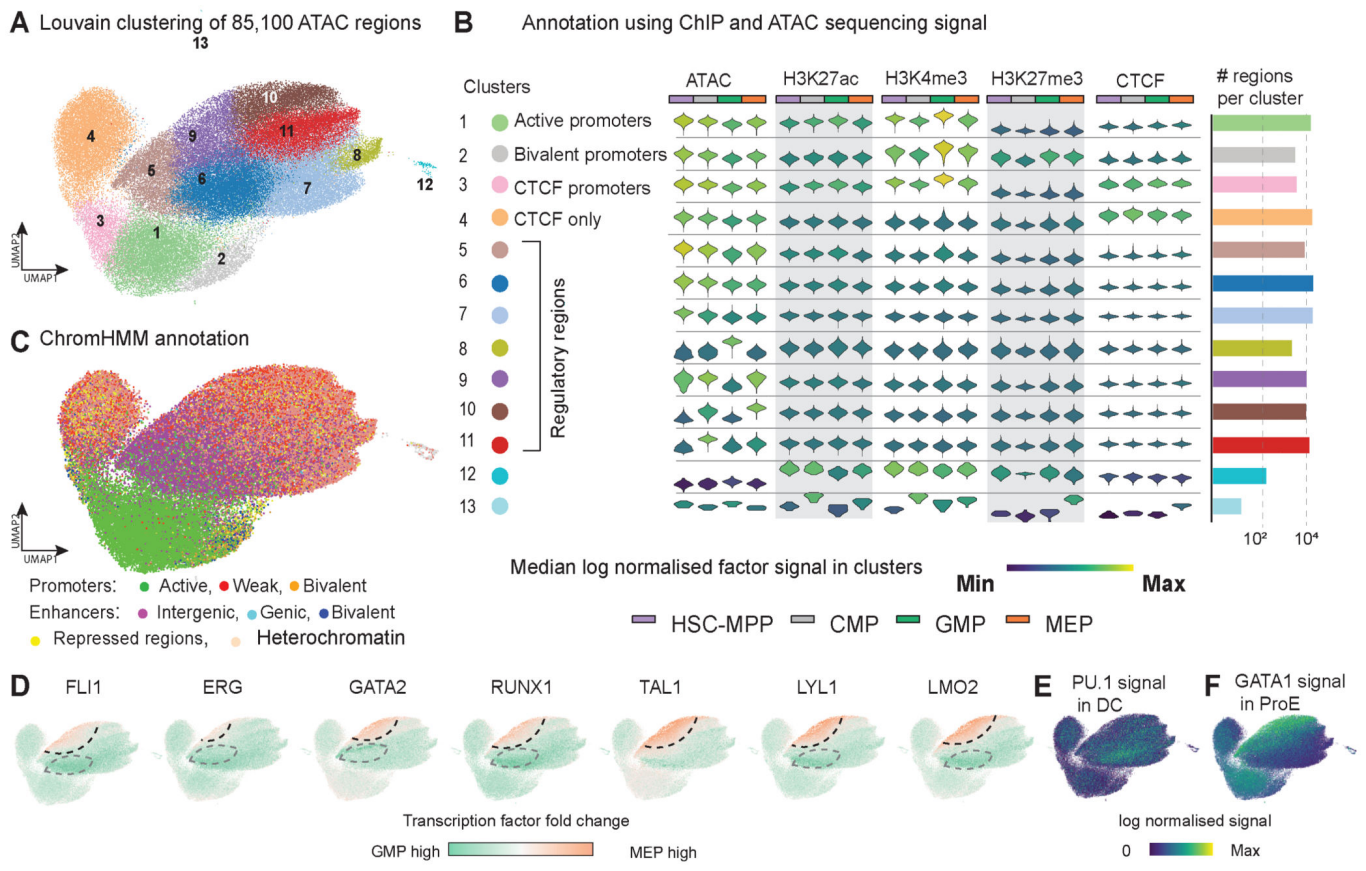
Regulatory regions with cell-type-specific heptad occupancy have distinct epigenetic features. **A)** A UMAP depicting the result of clustering 85,100 accessible regions in HSPCs annotated with ChIPmentation/ChIP-seq signal strengths using the Louvain algorithm. **B)** Individual violin plots of log normalised signal derived from ATAC, 3 histone marks (H3K27ac, H3K4me3, and H3K27me3), and CTCF − accompanied by a bar plot showing the number of regions in each cluster. Inter-cluster signal variability allows annotation of individual clusters based on their regulatory potential. **C)** UMAPs overlaid with ChromHMM annotation of 85,100 individual regions show striking similarity with **B**. **D)** UMAPs coloured based on log2 fold change of binding of the heptad transcription factors in pairwise comparisons between GMP and MEP. MEP- and GMP-specific enrichment of TF binding is identified, and borders demarcated by dashed lines: black (enriched in MEP) or grey (enriched in GMP). **E)** Signal of PU.1 in dendritic cells (DC) (GSE58864) across the clustered regions. PU.1 signal enrichment in dendritic cells mirrors heptad factor enrichment patterns in GMP. **F)** Signal of GATA1 in proerythroblasts (ProE) (GSE36985) across the clustered regions. GATA1 signal enrichment in proerythroblasts mirrors heptad factor enrichment patterns at these regions in MEP.

To better understand the underlying mechanisms regulating dynamic heptad occupancy during blood formation we used our ChIP datasets to annotate and cluster ~85000 regions previously shown to be accessible in any of our four cell types ^4^. The resulting UMAP projection of the accessibility landscape could be segmented into 13 clusters (Figure 6A). Clusters 1-3 had characteristics of promoters, including high H3K4me3 signal (Figure 6B). We could further classify cluster 1 as active promoters (H3K4me3 and H3K27ac), cluster 2 as bivalent promoters (H3K4me3 and H3K27me3 enriched), and cluster 3 as active promoters which were also bound by CTCF. These classifications aligned with ChromHMM annotation of the same regions (Figure 6C) ^52^. Cluster 4 was enriched for CTCF alone (Figure 6B) and may contain regions involved in 3D genome organization. Clusters 5-11 could be broadly characterized as non-promoter regulatory regions which again aligned with their ChromHMM annotations (Figure 6C). Most of the variations between these regions mapped to variable TF occupancy in specific cell types (Figure S11A), which was particularly pronounced in clusters 8-11 which also showed cell-type-specific changes in chromatin accessibility (Figure 6B). For example, cluster 9 and 10 showed high accessibility in MEP compared to GMP, accompanied by high TAL1 and LYL1 occupancy (Figure S11A), suggesting that regions in these clusters may function in the erythroid lineage.

Visualizing TF signal across the accessibility landscape revealed variable occupancy patterns across cell types (Figure S11B). FLI1, RUNX1, and ERG had similar distributions across all cell types, with highest FLI1 and RUNX1 occupancy in CMP and GMP, and ERG occupancy reduced in MEP. LYL1 and LMO2 had similar distributions with enrichment in clusters 5/6/7 in GMP and clusters 9/10 in MEP (Figure S11B). TAL1 was also enriched in clusters 9/10 in MEP with lower binding in other cell types, while PU.1 was enriched in cluster 5/6/7 in CMP and GMP (Figure S11B). GATA2 had a unique occupancy pattern with enrichment in cluster 5/6 in HSC-MPP and GMP and additional occupancy in cluster 9 in CMP and MEP (Figure S11B) which may reflect distinct roles of GATA2 in early hematopoiesis and subsequent erythroid specification ^53^.

We then made pairwise comparisons of TF binding signals in GMP vs MEP, HSC-MPP vs GMP, and HSC-MPP vs MEP (Figure 6D, Figure S11C). Comparing GMP (Figure 6D, green) to MEP (Figure 6D, orange), there were distinct zones of TF enrichment in each cell type (Figure 6D, grey dotted line [GMP], black dotted line [MEP]) except for TAL1 which showed no region of enrichment in GMP. To confirm that regions enriched for heptad TF binding in GMP and MEP represent lineage-specific regulators we mapped ChIP seq signal from lineage defining TFs in two mature cell types (Figure 6E, F). Regions with high heptad occupancy in GMP showed similar occupancy of PU.1 in dendritic cells (Figure 6E) ^54^, while regions with high heptad occupancy in MEP showed similar occupancy of GATA1 in proerythroblasts (Figure 6F)^6^. Taken together these data show that regulatory regions with heptad occupancy in progenitor populations are regions occupied by lineage-specific TFs in more mature cells.

### Cell-type specificity of regulatory elements is encoded in the underlying motif composition

**Figure 7:**
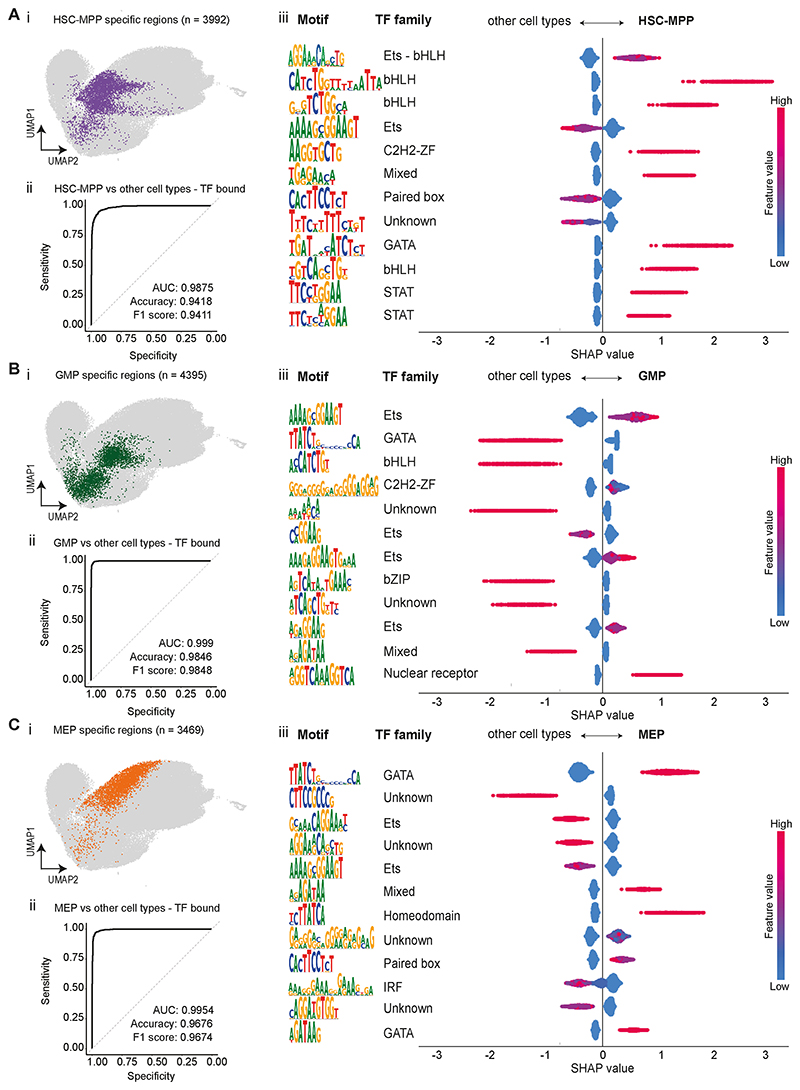
Cell-type specificity of regulatory elements is encoded in the underlying motif composition. **A)** (i) UMAP representation of ATAC-seq regions in CD34^+^ cells (grey) with heptad TF bound HSC-MPP specific regions colored in purple. (ii) An XGBoost machine learning model was trained and tested with motif counts from a mixture of regions specified in (i) and background regions, to predict cell type with high accuracy. The ROC curve shows the predictive performance of the constructed model to predict HSC-MPP specific regions. (iii) A beeswarm plot depicting the top 12 representative motifs in HSC-MPP specific regions - ranked based on their absolute importance in contributing to the predictive model. Each row shows the motif (and canonical TF family if known), and the corresponding SHAP (**SH**aply **A**dditive ex**P**lanations) values for the cell type in question (right) and the others (left). The feature count indicates the normalised motif counts with a range of 0-1. **B)** (i) UMAP representation of ATAC-seq regions in CD34^+^ cells (grey) with heptad TF bound GMP specific regions colored in green. (ii) ROC curve showing the performance of the model to predict GMP specific regions. (iii) A beeswarm plot depicting the top 12 representative motifs in GMP specific regions - ranked based on their absolute importance in contributing to the predictive model. **C)** (i) UMAP representation of ATAC-seq regions in CD34^+^ cells (grey) with heptad TF bound MEP specific regions colored in orange. (ii) ROC curve showing the performance of the model to predict MEP specific regions. (iii) A beeswarm plot depicting the top 12 representative motifs in MEP specific regions - ranked based on their absolute importance in contributing to the predictive model.

TFs bind DNA via consensus binding motifs whose sequence and relative locations in each regulator determine which TF complexes can potentially bind. To better understand enhancer features underpinning lineage-specific TF occupancy we selected regions with differential accessibility and TF occupancy in HSC-MPP (Figure 7Ai, 3992 regions), GMP (Figure 7Bi, 4395 regions) and MEP (Figure 7Ci, 3469 regions) and developed machine learning models to predict cell-type associations based on DNA sequence motifs. All models showed high sensitivity and specificity to associate regions with cell types (Figure 7Aii, Bii, Cii, Table S7). To understand how specific motifs contribute to assigning regulatory elements to cell types, we calculated SHapley Additive exPlanations (SHAP) values for each cell type (Figure 7Aiii, Biii, Ciii, Table S8). Distinct combinations of motifs were found to contribute to each model, many of whihc fit the expected profile for cell-specific regulatory regions. For example, ETS motifs had positive SHAP values in the GMP model but negative values in the MEP model, consistent with known roles for ETS factors such as PU.1 in driving myeloid differentiation, while GATA motifs had positive SHAP values in HSC-MPP and MEP models but negative values in the GMP model, consistent with GATA factors such as GATA1 driving the erythroid lineage ^55,56^. Motifs not corresponding to heptad factors had high SHAP scores in all models, consistent with heptad factors binding at lineage-specific enhancers in the context of larger regulatory protein complexes. We explored expression of non-heptad TFs corresponding to motifs predicted to positively impact SHAP scores for each model (Table S8). *GATA3* (HSC-MPP model), *PU.1* and *SPI-B* (GMP model) and *GATA1* (MEP model) had lineage-specific expression patterns consistent with known expression and activity of these factors ^53,56,49,57–60^ (Figure S12A-C). All other motif-associated-TFs had minimal expression variation across cell types.

Finally, we used our models to classify functional HSC regulatory elements identified through analysis of γ-retroviral integration sites (γRV-IS) in gene therapy patients ^61^. γRV-IS had similar features to HSC-MPP-specific regions (Figure S7A, Figure S12D). Importantly, our models scored γRV-IS as most likely to be HSC-MPP-specific, confirming the validity of our analyses (Figure S12E).

## Discussion

In this study we explored genome-wide dynamics of chromatin occupancy and structure in four cell types along the HSC to myeloid/erythroid differentiation axis. Analysis of putative CREs with direct looping to heptad promoter regions revealed greater regulatory complexity than previously appreciated. Previous work identified a set of nine regions with combinatorial heptad binding in human HSPCs whose relative accessibility is sufficient to predict cell identity ^23^. The current study identified more than 30 putative CREs directly looped to heptad promoters in at least one cell type (Figure 3B). While most previously described heptad regulatory elements have been tested in functional assays (Table S4), further validation is required to precisely understand individual and cooperative roles of specific CREs for each heptad gene ^62,63^. Surprisingly, some previously identified enhancers were not directly looped to heptad promoters in this analysis. For example *GATA2*−117, an enhancer dysregulated in inv(3) AML ^64,65^, was only indirectly looped to the *GATA2* promoter, and therefore excluded from our network model (Figure S6B). However, lack of direct binding does not preclude a role for this enhancer in regulating GATA2. Indeed, previous studies have found that enhancer − enhancer interactions can stabilise and amplify TF binding, and the resulting enhancer communities can drive and coordinate cell-type specific gene expression ^66,67^. Consistent with this model, we observed a trend of increasingly complex chromatin looping interactions involving heptad promoters and putative enhancers as cells committed to either erythroid or myeloid lineages (Figure 3B). Increased looping may restrict committed progenitors from accessing alternate fates or participate in shutting off stem cell programs. Intriguingly, the stem cell gene *ERG* ^30,23,29^ acquired loops to a +1200kb region in MEPs (Figure 3Ai), while increased long range looping and heptad binding occurred at the +45 enhancer of the erythroid gene *TAL1* ^68,14^ (Figure S6Di). Thus, increased looping can be associated with genes subsequently down- or up-regulated. Further experimentation is needed to fully understand rapidly increased chromatin looping in committed progenitors.

Motif-based analysis showed that heptad-occupied CREs contain information encoding cell-type-specificity. Among motifs that contributed to cell-type specificity we found motifs for additional factors including STAT, interferon-regulatory factor (IRF), and homeodomain TFs (Figure 7, Table S8). While our focus was on heptad factors, it is clear that additional regulators co-occupy CREs and contribute to enhancer specificity ^69^. To date these studies have focussed on transcriptional cofactors with broad function such as P53 and MED14 ^70^, and further work is required to understand how heptad factors, general cofactors, and intrinsic features of enhancers and promoters cooperate to regulate transcriptional programs throughout haematopoiesis.

Numerous bone marrow failure syndromes have an underlying germline genetic cause and may be amenable to gene therapy approaches targetting primitive HSCs to enable long term benefits. However, ectopic gene expression may lead to lineage skewing and other unwanted outcomes. Cell-type-specific enhancers are conserved across evolution and can potentially be used to restrict transgene expression ^71,72,6^. Indeed, erythroid-specific *GATA1* enhancers can drive restricted expression of GATA1 to rescue erythroid differentiation in Diamond-Blackfan Anaemia ^73^. However, identifying enhancers with appropriate spatiotemporal activity is not trivial. While experimental techniques for massively parallel interrogation of enhancer activity and specificity have been developed ^74,63^, our data provides a catalogue of putative enhancers that could fast track functional studies.

GRNs are significantly perturbed in multiple AML subtypes ^75,8^, often as a result of specific translocation events including RUNX1-RUNX1T1 ^76,77^and inv(3) ^65,64^. Recent sequencing studies found that small structural variants (SVs) can mediate enhancer hijacking and subsequent oncogenic transformation ^63,78^. However, understanding the effects of SVs requires significant knowledge of cell-specific enhancers; our data set provides extensive annotation of regulatory regions that could be used to guide functional experiments exploring the effects of specific SVs.

While extensive, our dataset is limited to myeloid cell types. Sorted HSPCs, particularly CMPs, are also inherently heterogenous ^79–81^ which may limit our ability to resolve differences between adjacent cell types. Furthermore, while capable of reconstituting transplanted patients, mobilized cells may have subtle epigenomic differences to their unstimulated counterparts. Finally, even using low cell input techniques, our datasets represent averaged binding and looping probabilities captured across a shifting epigenetic landscape, rather than truly capturing GRNs, which operate at the level of single cells ^7^. Nonetheless, our dataset represents the most extensive epigenetic characterisation of primary human blood progenitors to date and provides important insights for future studies.

## Supplementary Material

Supplementary Materials

## Key Points

* Heptad TF occupancy is highly dynamic across HSPC subsets and associated with cell-type specific gene expression.
* Enhancers with cell-type-specific Heptad occupancy share a common grammar with respect to TF binding motifs.
