# Supplementary material for: Genome-wide Transcription Factor binding maps reveal cell-specific changes in the regulatory architecture of human HSPC
Source: Blood. Author manuscript; Available in PMC 2024 Apr 11. (PMC10651876; doi:10.1182/blood.2023021120)
Supplement: Supplementary Materials [file EMS185458-supplement-Supplementary_Materials.pdf]

Subramanian et al Supplemental Tables and Figures, and Methods

List of supplemental Tables:

Table S1. Donor information

Table S2. ChIP data mapping summary

Table S3. Significant HiChIP loops

Table S4. List of heptad *cis* Regulatory Elements (CREs)

Table S5. Gene lists used in Figure 4 and Figure 5

Table S6. Complete statistics for Supplemental Figure 9

Table S7. Model performance

Table S8. Motifs

Table S9. Key reagents used in this work

Table S10. Indexing primers

Supplemental Figure Titles and Legends

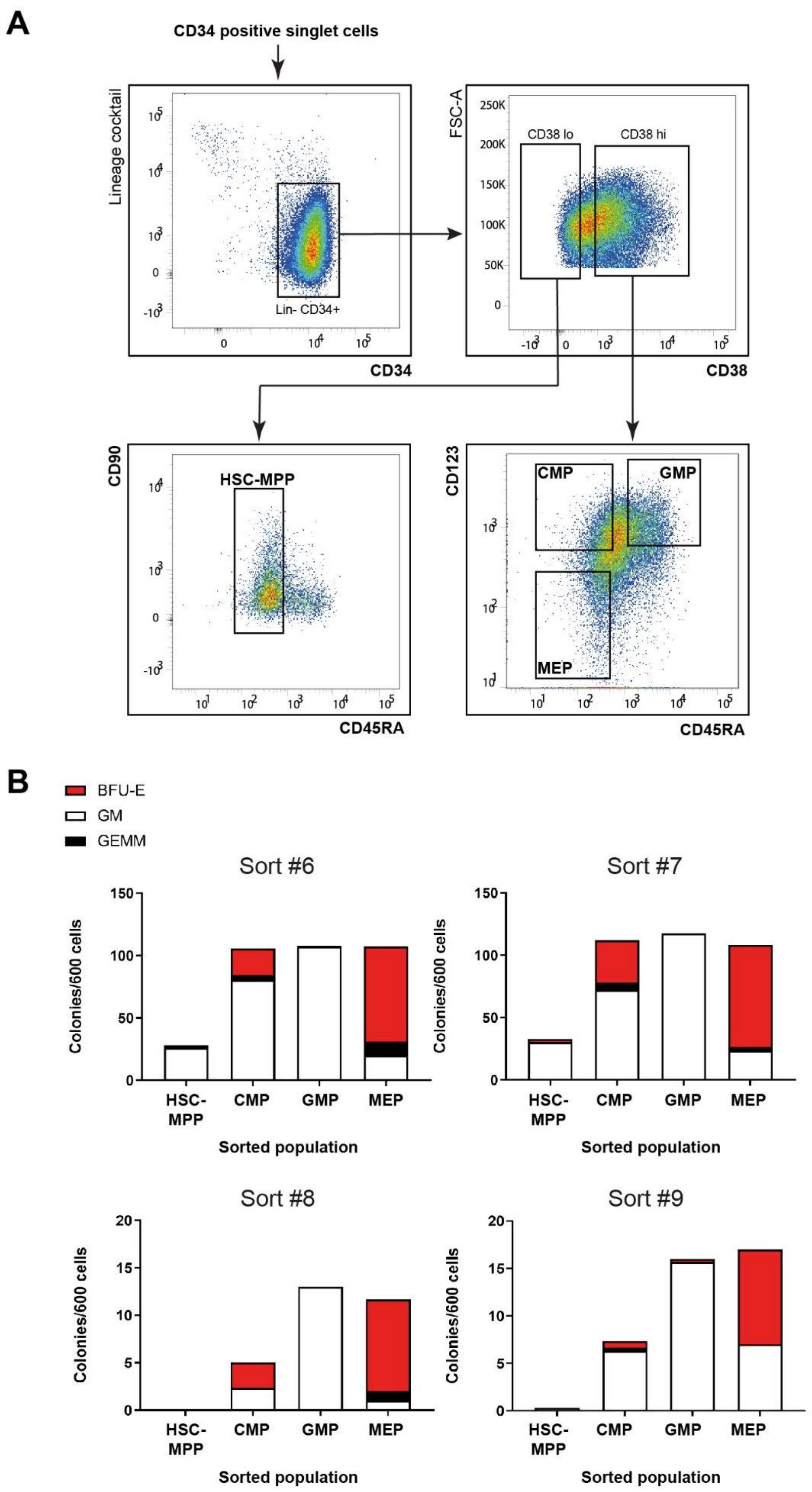
A
CD34 positive singlet cells
Lineage cocktail
CD34
Lin- CD34+
FSC-A
CD38
CD38 lo
CD38 hi
CD90
CD45RA
HSC-MPP
CD123
CMP
GMP
MEP
B
BFU-E
GM
GEMM
Sort #6
Sort #7
Sort #8
Sort #9
Colonies/600 cells
HSC-MPP
CMP
GMP
MEP
Sorted population

Figure S1

Figure S1. ***Workflow for deriving HSPC fractions and verifying their identity*.** **A)** Gating strategy and representative sort gates for isolation of HSC-MPP, CMP, GMP, and MEP. **B)** Representative CFU assays using sorted HSC-MPP, CMP, GMP, and MEP populations.

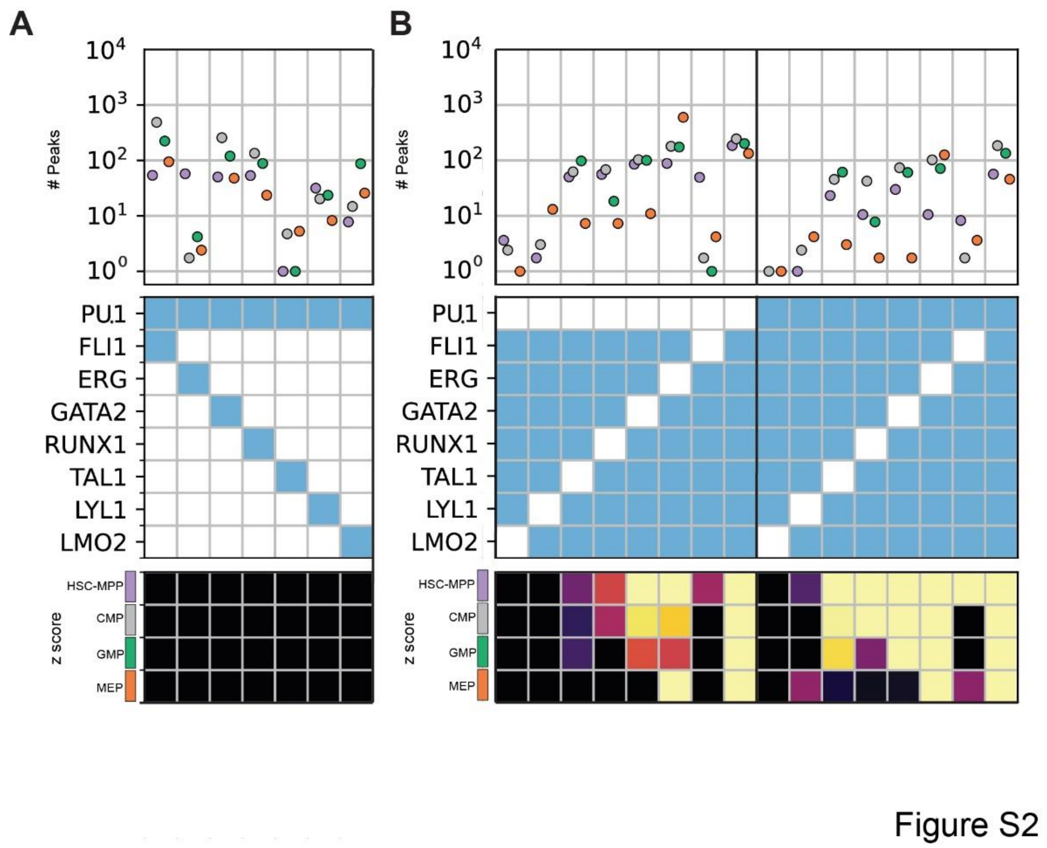

Figure S2. ***Combinatorial binding of heptad transcription factors and PU.1.*** **(A-B)** composite graphs with three components; *(upper)* number of combinatorial binding peaks identified in the four cell types, for *(middle)* combinations of heptad factors and PU.1 and *(lower)* heatmap showing z-scores for the combinations presented above. **A)** 2 TF combinations including PU.1. **B)** 6, 7, 8 TF combinations of heptad TFs without and with PU.1.

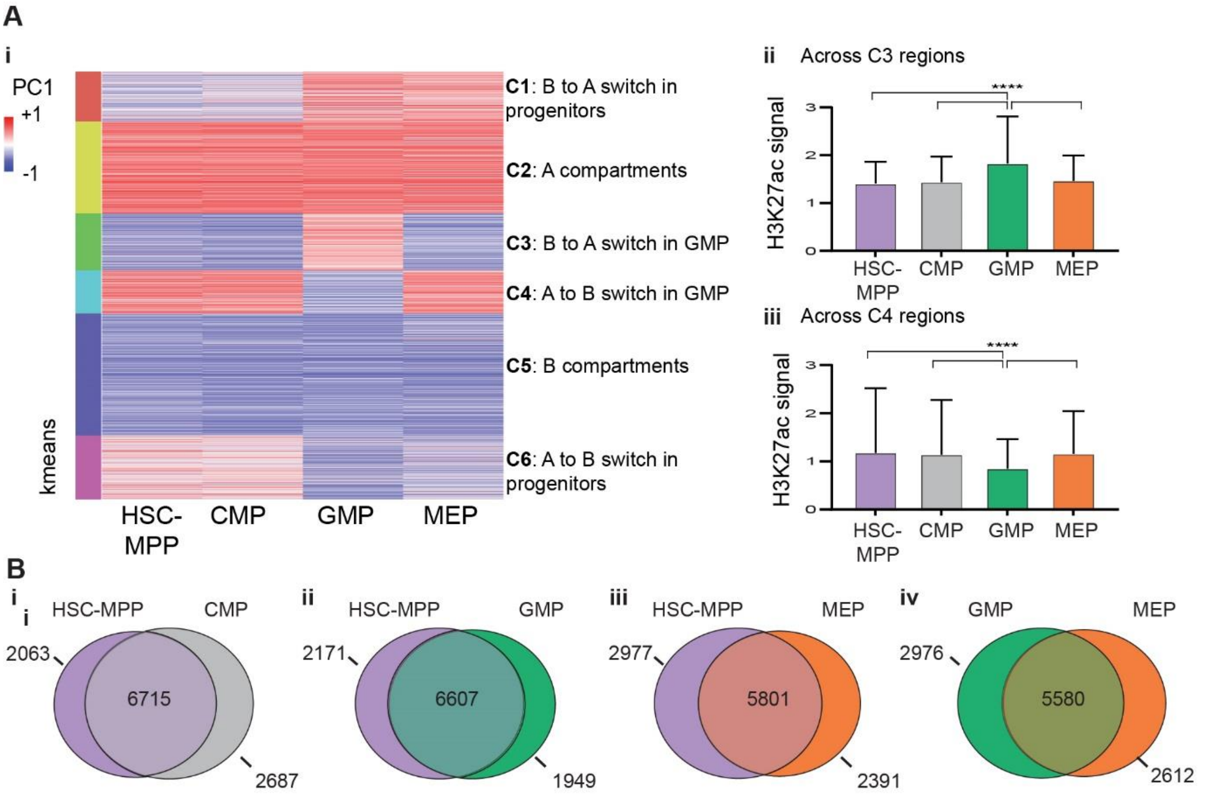

Figure S3

Figure S3. *Compartment switching and conserved TADs along blood stem cell differentiation*. A) i) A *k*-means clustered heatmap of the PC1 values showing compartmental switches taking place between the four HSPC populations. Average H3K27ac signal in ii) Cluster C3 regions, and in iii) Cluster C4 regions among the four cell types. Significance scores were calculated using pairwise t tests ($p < 0.0001$). **B)** Pairwise comparisons of topological domain (TAD) boundaries between i) HSC-MPP and CMP, ii) HSC-MPP and GMP, iii) HSC-MPP and MEP, and iv) GMP and MEP (domains identified using HOMER).

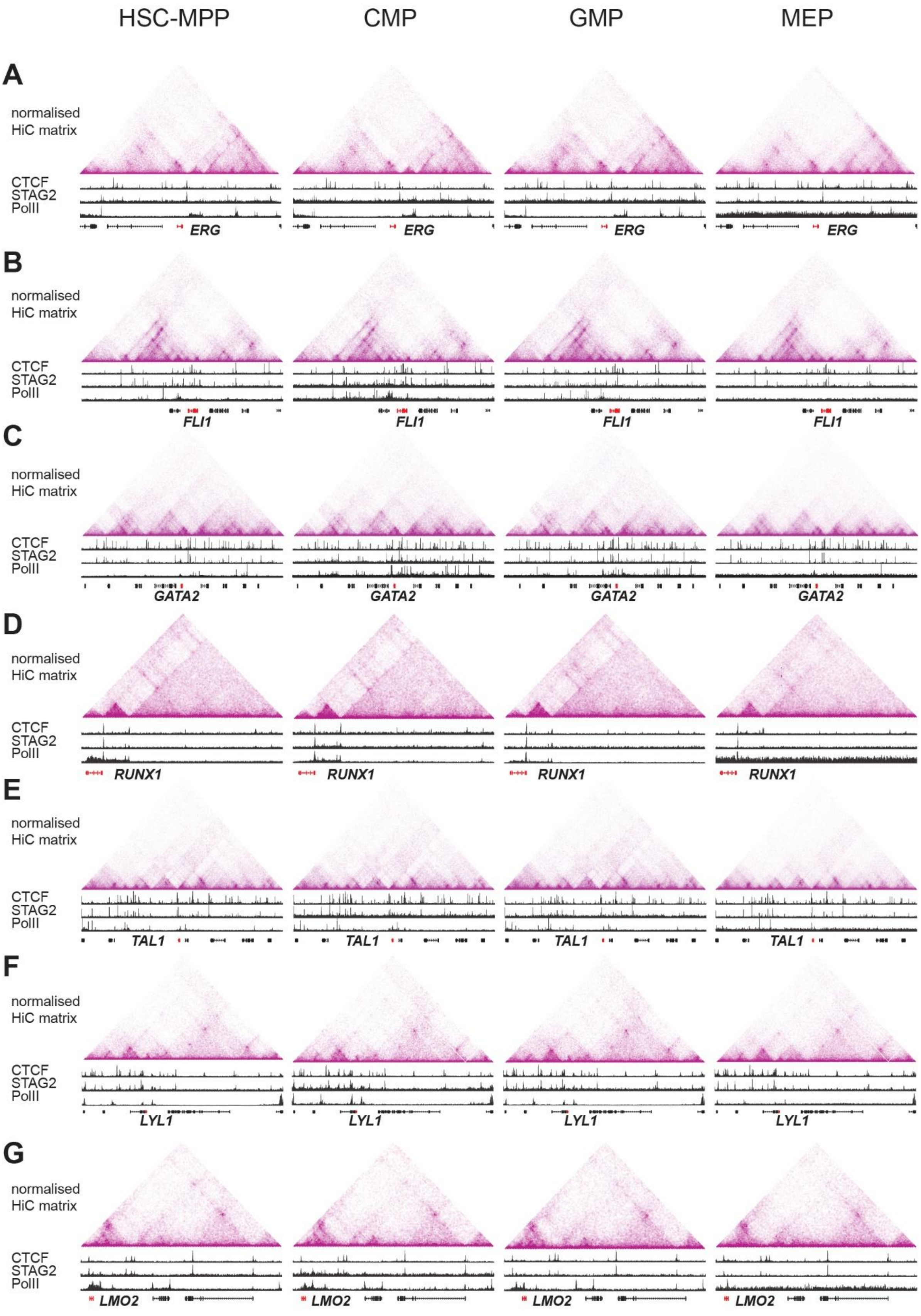

HSC-MPP
CMP
GMP
MEP
A
normalised HiC matrix
CTCF
STAG2
PolII
ERG
B
FLI1
C
GATA2
D
RUNX1
E
TAL1
F
LYL1
G
LMO2

Figure S4. ***The genome architecture at heptad gene regulatory loci is conserved across HSPC subsets.*** Normalised HiC contact matrices at 10 kb resolution located at individual heptad genes' regulatory loci – **A)** *FLI1* locus (GRCh38 chr11:128511084-128978507), **B)** *ERG* locus (GRCh38 chr21:37370238-39198738), **C)** *GATA2* locus (GRCh38 chr3:128262936-128761435), **D)** *RUNX1* locus (GRCh38 chr21:34758869-36011624), **E)** *TAL1* locus (GRCh38 chr1:47168881-47340728), **F)** *LYL1* locus (GRCh38 chr19:12787014-13852204), and **G)** *LMO2* locus (GRCh38 chr11:33831641-34445745), in HSC-MPP, CMP, GMP, and MEP respectively. Accompanying each triangular plot are ChIP-seq tracks showing normalized CTCF, STAG2, and PolII signal.

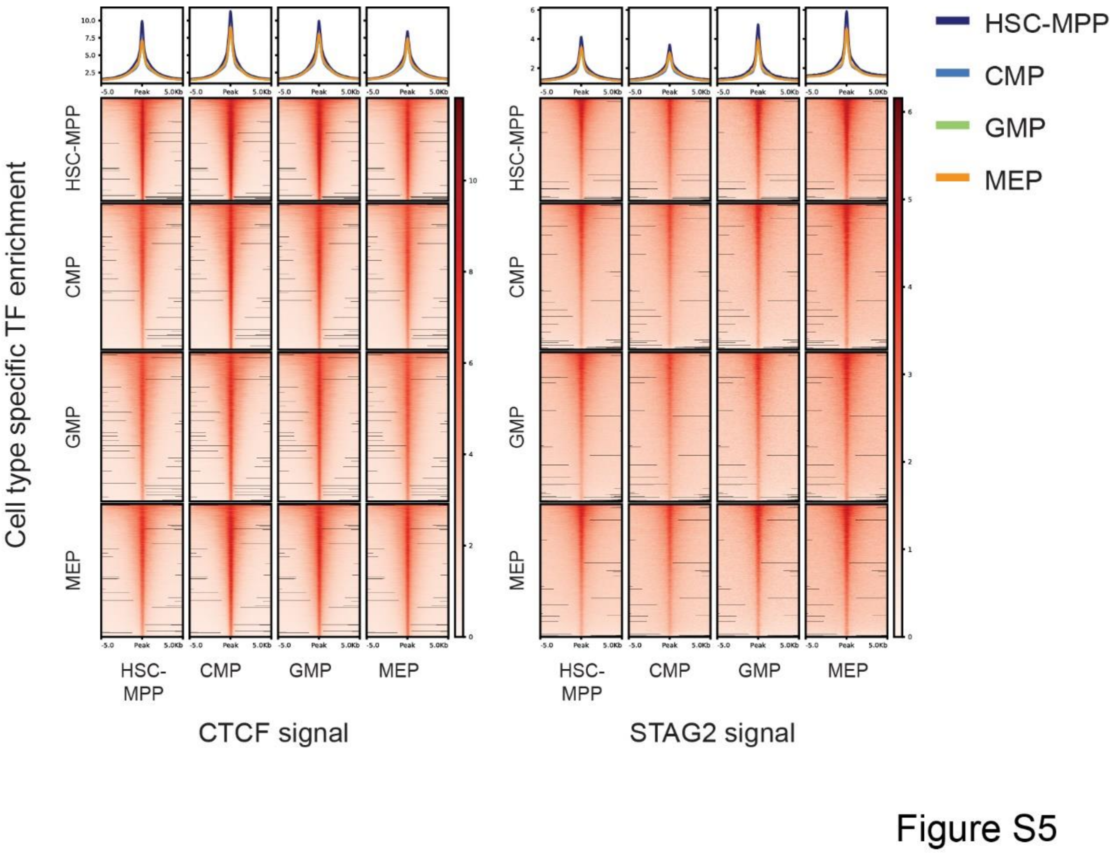

Figure S5. ***CTCF and STAG2 occupancy across cell types.*** Density plots showing CTCF (*left*) and STAG2 (*right*) signal in each cell type at CTCF peak regions (identified by macs2; p <1e-5) in HSC-MPP, CMP, GMP, MEP. CTCF and STAG2 occupancy at CTCF-bound sites shows minimal variation between cell types.

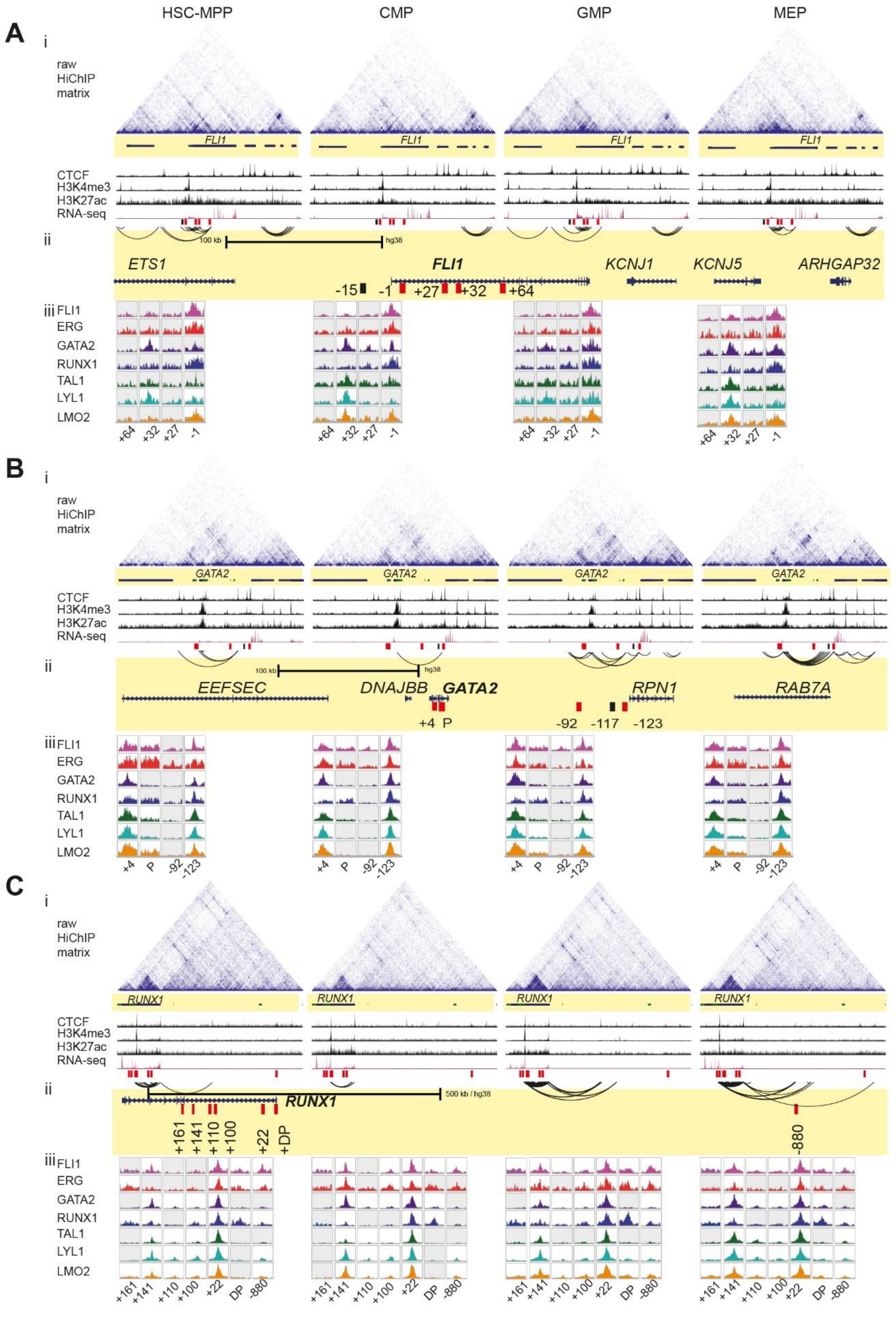
HSC-MPP
CMP
GMP
MEP
A
i
raw HiChIP matrix
FLI1
CTCF
H3K4me3
H3K27ac
RNA-seq
ii
100 kb
hg38
ETS1
FLI1
KCNJ1
KCNJ5
ARHGAP32
-15
-1
+27
+32
+64
iii
FLI1
ERG
GATA2
RUNX1
TAL1
LYL1
LMO2
+64
+32
+27
-1
B
i
raw HiChIP matrix
GATA2
CTCF
H3K4me3
H3K27ac
RNA-seq
ii
100 kb
hg38
EEFSEC
DNAJBB
GATA2
RPN1
RAB7A
+4
P
-92
-117
-123
iii
FLI1
ERG
GATA2
RUNX1
TAL1
LYL1
LMO2
+4
P
-92
-123
C
i
raw HiChIP matrix
RUNX1
CTCF
H3K4me3
H3K27ac
RNA-seq
ii
500 kb / hg38
RUNX1
+161
+141
+110
+100
+22
+DP
-880
iii
FLI1
ERG
GATA2
RUNX1
TAL1
LYL1
LMO2
+161
+141
+110
+100
+22
DP
-880

Figure S6

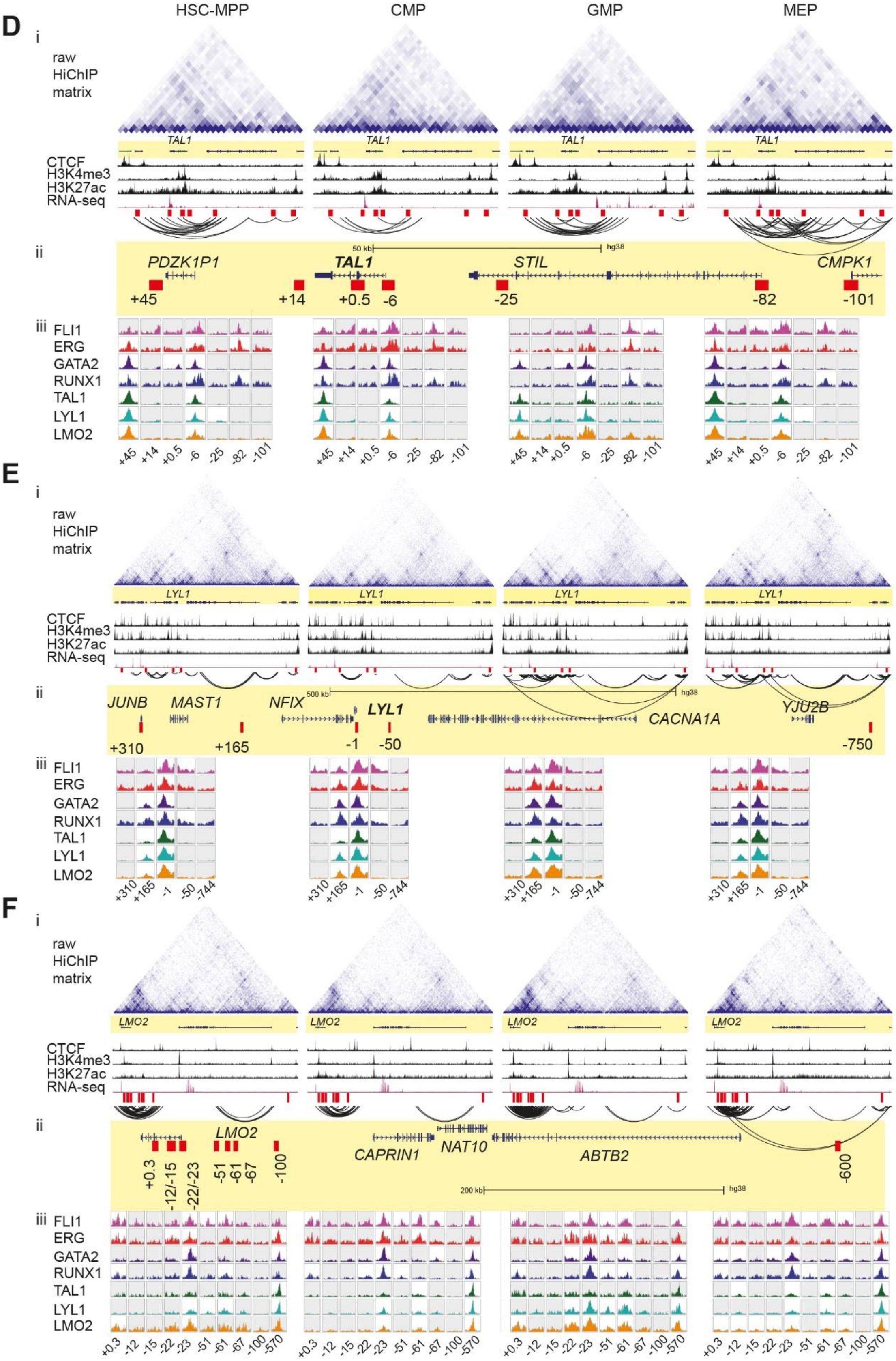
D
HSC-MPP
CMP
GMP
MEP
i
raw HiChIP matrix
TAL1
CTCF
H3K4me3
H3K27ac
RNA-seq
ii
PDZK1P1
TAL1
STIL
CMPK1
+45
+14
+0.5
-6
-25
-82
-101
iii
FLI1
ERG
GATA2
RUNX1
TAL1
LYL1
LMO2
E
i
raw HiChIP matrix
LYL1
CTCF
H3K4me3
H3K27ac
RNA-seq
ii
JUNB
MAST1
NFIX
LYL1
CACNA1A
YJU2B
+310
+165
-1
-50
-750
iii
FLI1
ERG
GATA2
RUNX1
TAL1
LYL1
LMO2
+310
+165
-1
-50
-744
F
i
raw HiChIP matrix
LMO2
CTCF
H3K4me3
H3K27ac
RNA-seq
ii
LMO2
CAPRIN1
NAT10
ABTB2
+0.3
-12/-15
-22/-23
-51
-61
-67
-100
-600
iii
FLI1
ERG
GATA2
RUNX1
TAL1
LYL1
LMO2
+0.3
-12
-15
-22
-23
-51
-61
-67
-100
-570

Figure S6

Figure S6. ***H3K27ac HiChIP identifies cell-type specific interactions between heptad gene promoters and potential regulatory regions.*** **A)** i) Raw HiChIP contact matrix, CTCF, H3K4me3, H3K27ac, IgG, RNA-seq, and significant H3K27ac HiChIP interactions (FDR ≤ 0.01) at the *FLI1* locus (GRCh38 chr11:128511084-128978507). ii) Magnified view of the *FLI1* locus, with potential regulators looping to the promoter shown in red and potential regulators engaged in indirect regulatory activities shown in black. iii) FLI1, ERG, GATA2, RUNX1, TAL1, LYL1, and LMO2 peaks at the regulatory regions defined in ii, are shown. **B)** i) Raw HiChIP contact matrix, CTCF, H3K4me3, H3K27ac, IgG, RNA-seq, and significant H3K27ac HiChIP interactions (FDR ≤ 0.01) at the *GATA2* locus (chr3:128,262,936-128,761,435). ii) Magnified view of the *GATA2* locus, with potential regulators looping to the promoter shown in red and potential regulators engaged in indirect regulatory activities shown in black. iii) FLI1, ERG, GATA2, RUNX1, TAL1, LYL1, and LMO2 peaks at the regulatory regions defined in ii, are shown. **C)** i) Raw HiChIP contact matrix, CTCF, H3K4me3, H3K27ac, IgG, RNA-seq, and significant H3K27ac HiChIP interactions (FDR ≤ 0.01) at the *RUNX1* locus (GRCh38 chr21:34758869-36011624). ii) Magnified view of the *RUNX11* locus, with potential regulators looping to the promoter shown in red. iii) FLI1, ERG, GATA2, RUNX1, TAL1, LYL1, and LMO2 peaks at the regulatory regions defined in ii, are shown. **D)** i) Raw HiChIP contact matrix, CTCF, H3K4me3, H3K27ac, IgG, RNA-seq, and significant H3K27ac HiChIP interactions (FDR ≤ 0.01) at the *TAL11* locus (GRCh38 chr1:47,168,881-47,340,728). ii) Magnified view of the *TAL11* locus, with potential regulators looping to the promoter shown in red. iii) FLI1, ERG, GATA2, RUNX1, TAL1, LYL1, and LMO2 peaks at the regulatory regions defined in ii, are shown. **E)** i) Raw HiChIP contact matrix, CTCF, H3K4me3, H3K27ac, IgG, RNA-seq, and significant H3K27ac HiChIP interactions (FDR ≤ 0.01) at the *LYL1* locus (GRCh38 chr19:12787014-13852204). ii) Magnified view of the *LYL1* locus, with potential regulators looping to the promoter shown in red. iii) FLI1, ERG, GATA2,

RUNX1, TAL1, LYL1, and LMO2 peaks at the regulatory regions defined in ii, are shown. **F)** i) Raw HiChIP contact matrix, CTCF, H3K4me3, H3K27ac, IgG, RNA-seq, and significant H3K27ac HiChIP interactions (FDR $\leq$ 0.01) at the *LMO2* (GRCh38 chr11:33831641-34445745). ii) Magnified view of the *LMO2* locus, with potential regulators looping to the promoter shown in red. iii) FLI1, ERG, GATA2, RUNX1, TAL1, LYL1, and LMO2 peaks at the regulatory regions defined in ii, are shown.

Only those HiChIP interactions where both interacting ends were found at the given locus are shown. In addition the ChIP-seq peaks shown are RPKM-normalised and white boxes indicate presence of a computationally called ChIP-seq peak at the specific region.

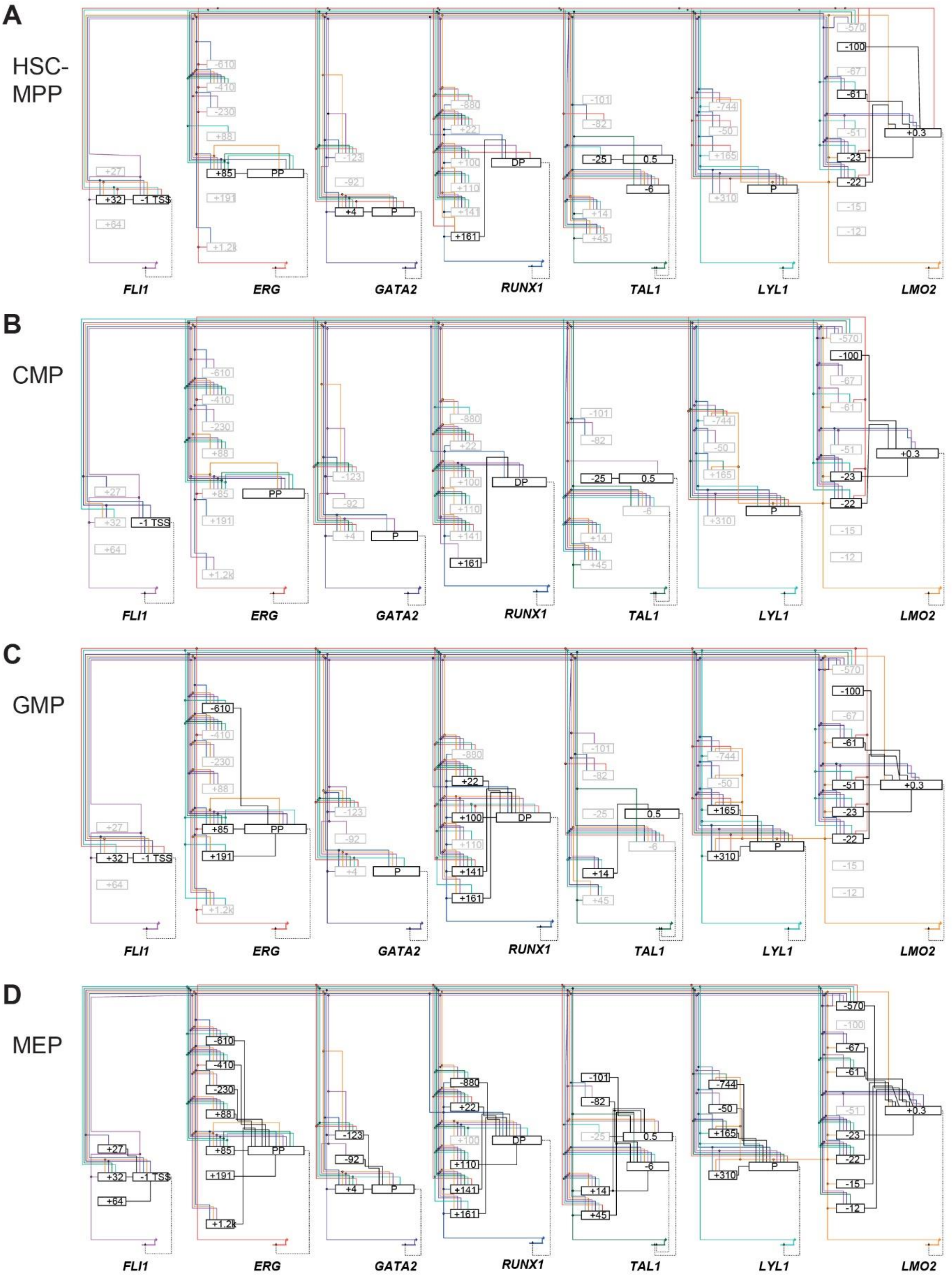

Figure S7

Figure S7. ***Gene regulatory network maps of the heptad genes in HSPC subsets*.** Heptad GRNs in **A)** HSC-MPP, **B)** CMP, **C)** GMP, and **D)** MEP, constructed using BioTapestry software. Boxes in bold show active regulators, and their interaction with respective promoters marked with solid black lines. Solid coloured lines indicate heptad factors binding to regulatory regions (FLI1-pink, ERG-red, GATA2-purple, RUNX1-dark blue, TAL1-green, LYL1-aqua, and LMO2-orange), while dashed lines link regulatory sub-circuits to individual genes.

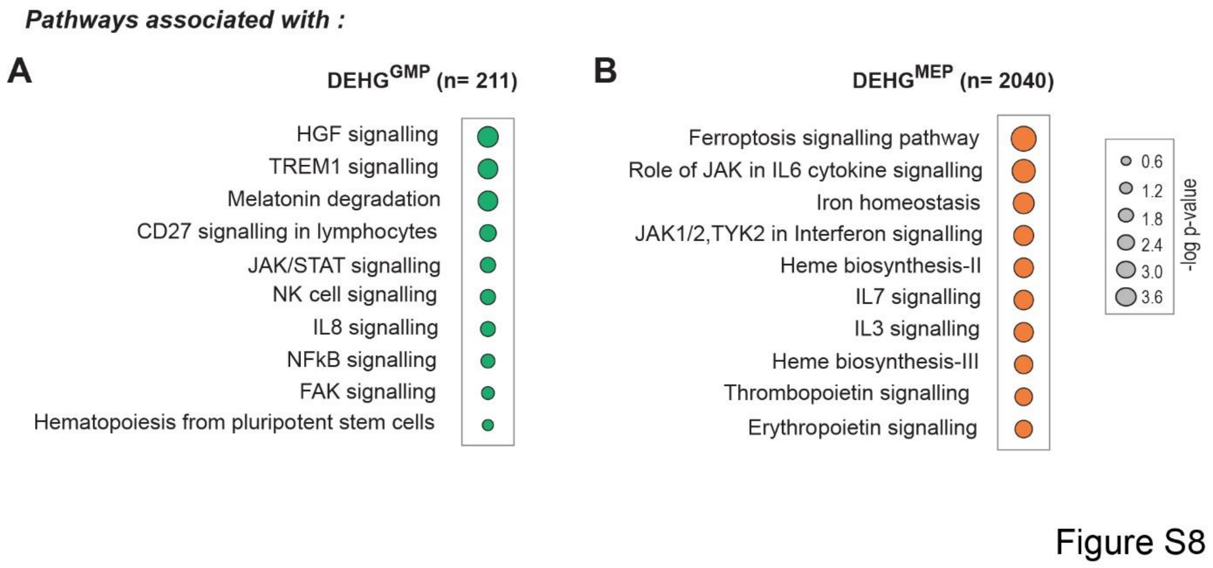

Figure S8. ***The role of heptad transcription factors in regulating lineage-specific gene expression.*** Ingenuity pathway analysis performed in DEHGs: **A)** $DEHG^{GMP}$, and **B)** $DEHG^{MEP}$.

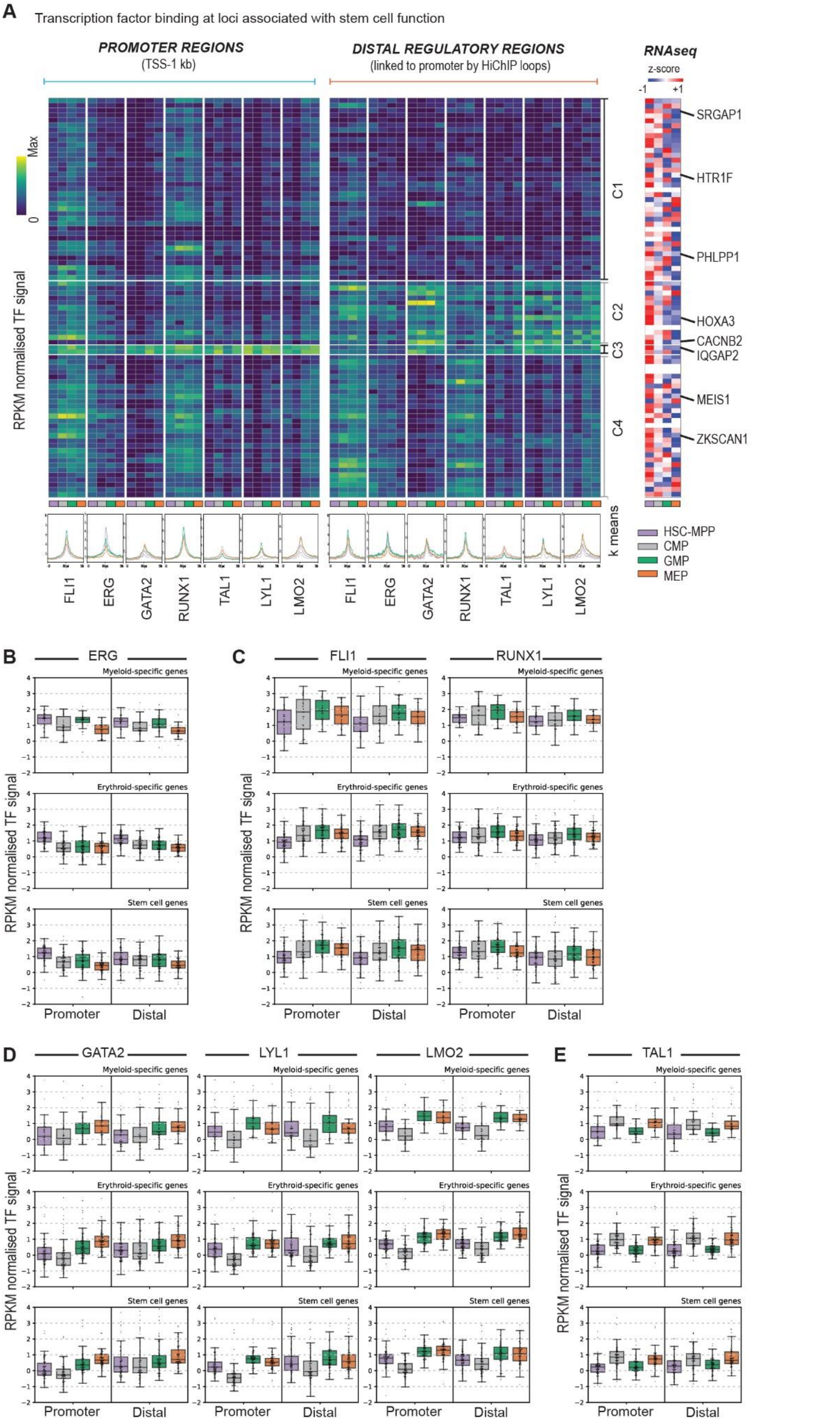

A
Transcription factor binding at loci associated with stem cell function
PROMOTER REGIONS
(TSS-1 kb)
DISTAL REGULATORY REGIONS
(linked to promoter by HiChIP loops)
RNAseq
z-score
-1
+1
Max
0
RPKM normalised TF signal
C1
C2
C3
C4
k means
SRGAP1
HTR1F
PHLPP1
HOXA3
CACNB2
IQGAP2
MEIS1
ZKSCAN1
HSC-MPP
CMP
GMP
MEP
FLI1
ERG
GATA2
RUNX1
TAL1
LYL1
LMO2
B
ERG
C
FLI1
RUNX1
Myeloid-specific genes
Erythroid-specific genes
Stem cell genes
Promoter
Distal
D
GATA2
LYL1
LMO2
E
TAL1

Figure S9

Figure S9. ***Cell-type specific binding patterns of heptad factors identified across stem cell specific genes***. **A)** Genes associated with stem cell function. *Left:* k-means clustered heatmaps of TF binding intensity at promoters and distal regulatory regions. Profile plots show normalised signal for each TF in each cell type at the regions depicted in the heatmap. *Right:* z-score normalised heatmaps of RNA-seq counts (GSE75384) for the corresponding gene in each cell type. **B-E)** Normalised TF signal at all promoters and distal regulatory regions for myeloid, erythroid, and stem cell genes. P-values for all pairwise comparisons (paired t-test) are shown in Table S4. **B)** Boxplots showing normalised ERG signal at promoters and distal regulatory regions of myeloid, erythroid, and stem cell genes. **C)** Boxplots showing normalised FLI1 and RUNX1 signal at promoters and distal regulatory regions of myeloid, erythroid, and stem cell genes. **D)** Boxplots showing normalised GATA2, LYL1, and LMO2 signal at promoters and distal regulatory regions of myeloid, erythroid, and stem cell genes. **E)** Boxplots showing normalised TAL1 signal at promoters and distal regulatory regions of myeloid, erythroid, and stem cell genes.

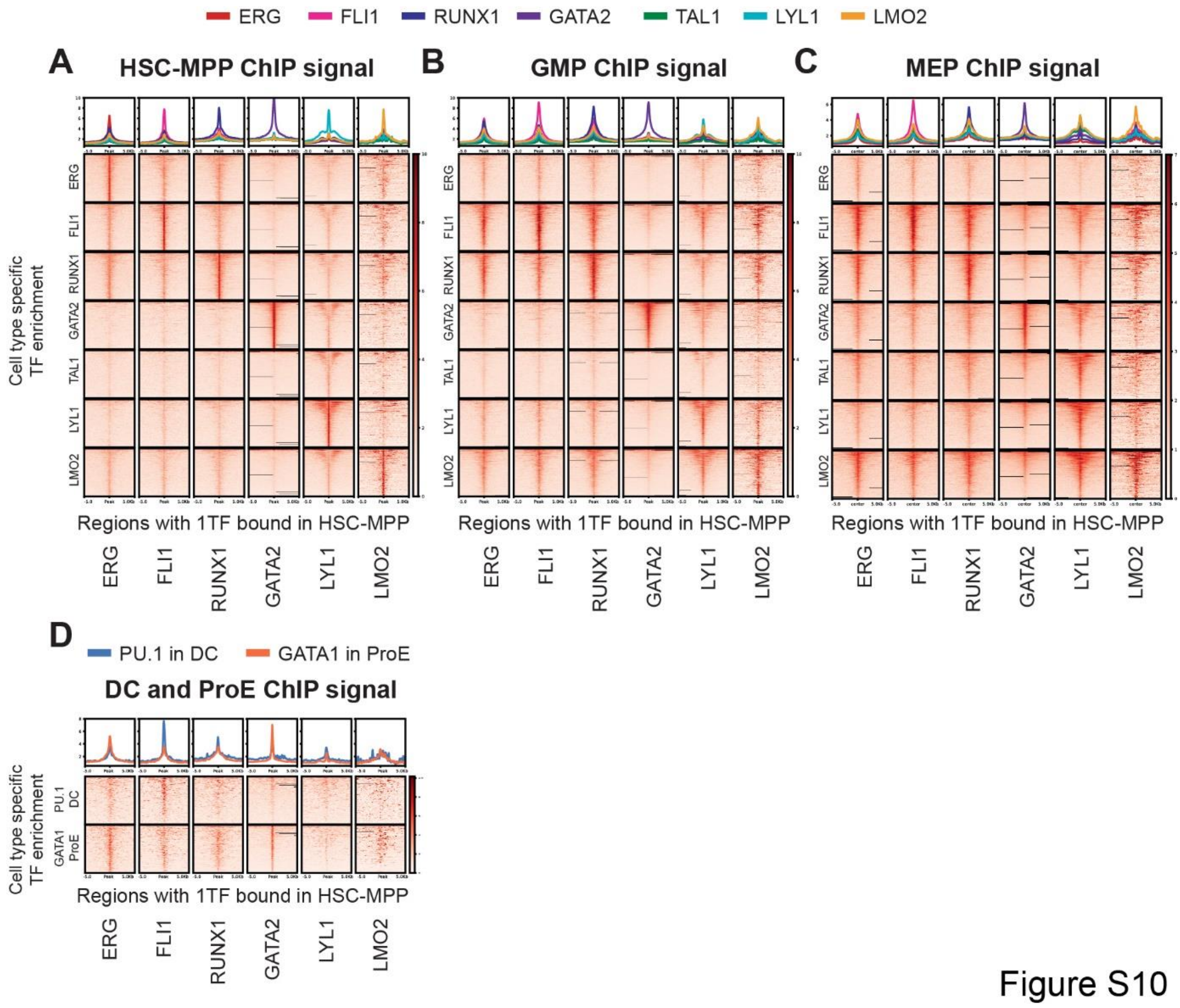

Figure S10. ***Sites bound by single heptad factors in HSC-MPPs are commonly bound by additional heptad factors in committed progenitors and mature cells.*** **A-C)** Density plots showing enrichment of TFs in **A)** HSC-MPPs **B)** GMPs **C)** MEPs at regions called as peaks for one heptad factor in HSC-MPP. TAL1 had only 54 peaks in HSC-MPPs which precluded analysis using this method. Columns show peak regions that are unique to the indicated heptad factor in HSC-MPP, rows show signal density of the indicated TF across those regions. **D)** Density plots showing enrichment of PU.1 in dendritic cells (DC) (GSE58864) and GATA1 in proerythroblasts (ProE) (GSE36985) at regions called as peaks for one heptad factor in HSC-MPP.

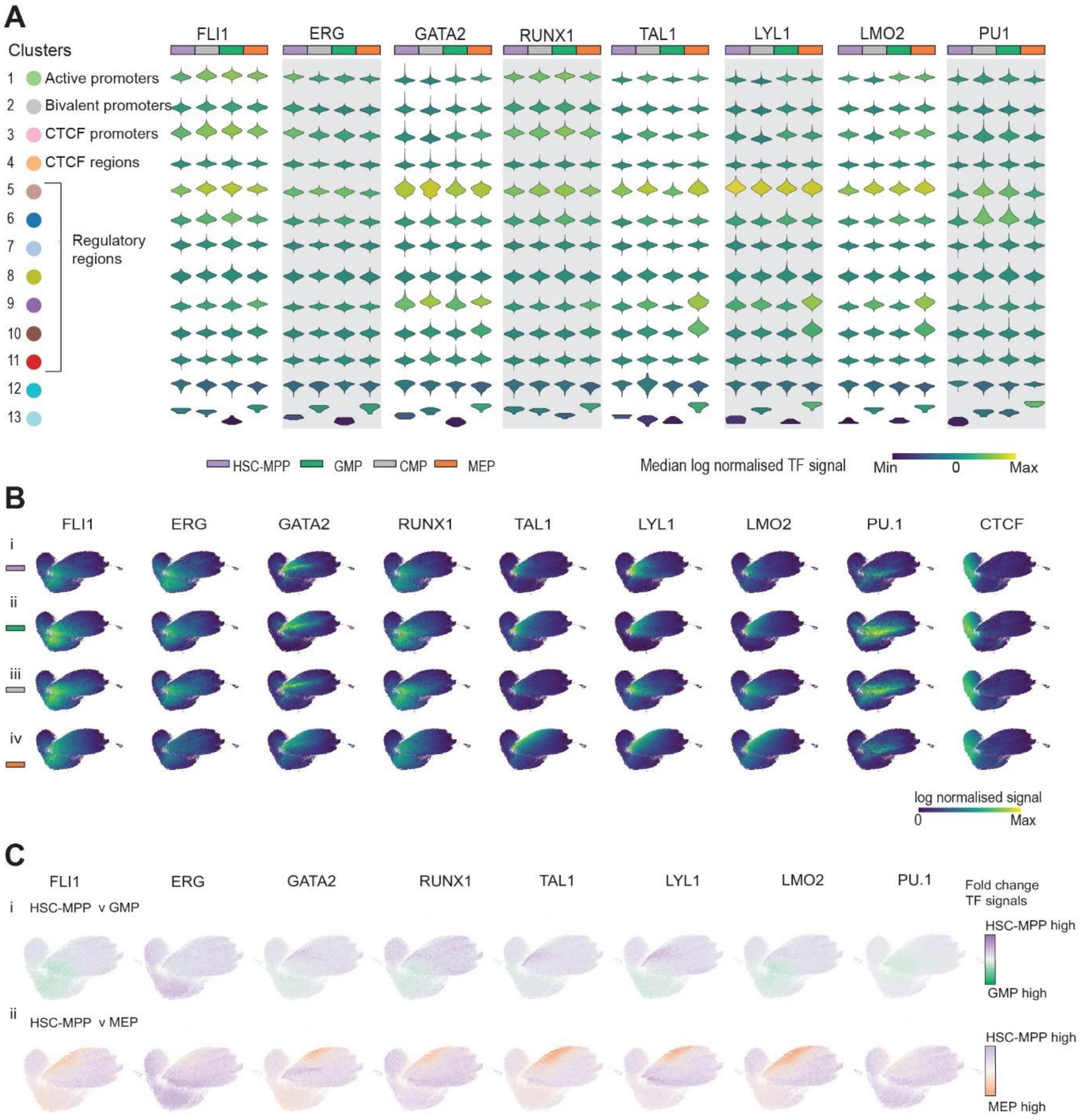

Figure S11

Figure S11. ***Transcription factor signal enrichment at ATAC regions in HSPCs.*** **A)** Individual violin plots show normalized transcription factor signals for FLI1, ERG, GATA2, RUNX1, TAL1, LYL1, and LMO2, in each of the 13 derived clusters for the four cell types studied. **B)** UMAPs showing heptad-factor-, PU.1-, and CTCF-normalized signals at accessible regions in i) HSC-MPP, ii) CMP, iii) GMP, and iv) MEP. **C)** UMAPs colored based on log2

fold change of binding of heptad transcription factors and PU.1 in pairwise comparisons between i) HSC-MPP and GMP, and ii) HSC-MPP and MEP.

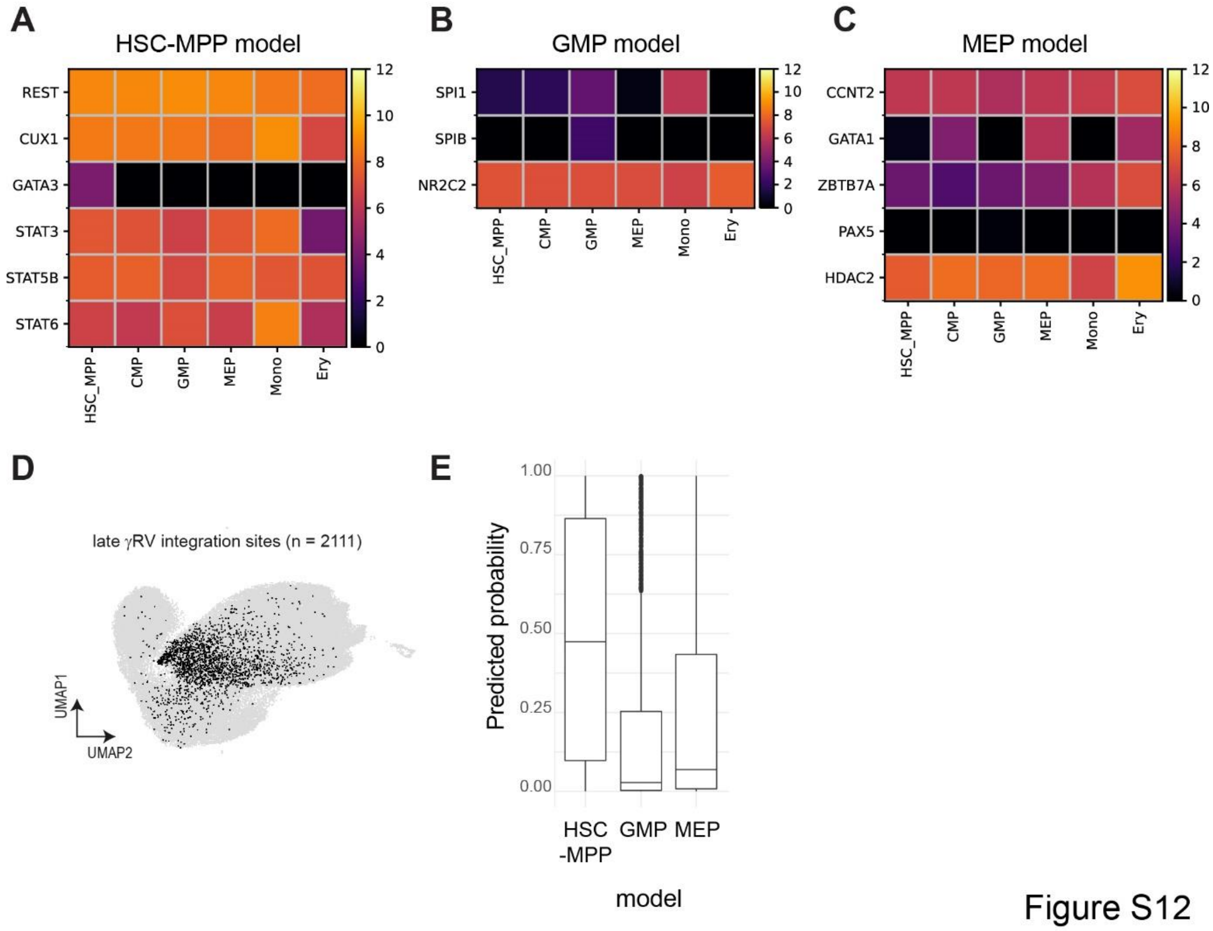

Figure S12. ***Cell-type specificity of regulatory elements is encoded in the underlying motif composition*.** **(A-C)** Heatmaps showing cpm-normalised RNA-seq counts (GSE75384) for selected genes in HSC-MPPs, CMPs, GMPs, MEPs, monocytes (Mono), erythroblasts (Ery). Genes shown correspond to motifs with positive SHAP values (Figure 7) for **A)** HSC-MPP model, **B)** GMP model, **C)** MEP model. Only genes expressed in at least one cell type are shown. **D)** UMAP representation of ATAC-seq regions in $CD34^+$ cells (grey) with regions corresponding to late γ retroviral integration sites (γRV-IS, n = 2111) colored in black. Late γRV-IS integration sites correspond to functionally defined regulatory regions in long term repopulating human HSCs [1]. **E)** Graphs showing predicted probabilities that γRV-IS are HSC-MPP-, GMP-, or MEP-specific for n = 1674 γRV-IS. Sites that overlapped the model training data were excluded from prediction analysis.

Supplemental Methods

Antibodies, key reagents, and software

A complete list of key reagents is available in Table S9.

Biological samples

Mobilized peripheral blood samples were collected with patient consent in accordance with the Declaration of Helsinki, and used with institutional ethics approval ref:08/190 from South Eastern Sydney Local Health District, NSW, Australia.

Isolation of $CD34^+$ cells from cryopreserved apheresis packs

Cryopreserved cells were thawed, diluted 1:4 in 2.5% dextran/12.5% human albumin in 0.9% saline, centrifuged 200g/15 min/room temperature (RT) and resuspended in phosphate-buffered saline (DPBS) containing 10% fetal bovine serum (FBS). Cells were underlaid with lymphoprep, centrifuged 800g/30 min/RT, and mononuclear cells (MNCs) collected from the interface and washed with DPBS. MNCs were resuspended in ice cold CliniMACS buffer supplemented with 0.5% human albumin or AutoMACS running buffer then labelled with anti-CD34 microbeads according to manufacturer's instructions (Miltenyi Biotec). $CD34^+$ cells were enriched using either a CliniMACS Plus (Miltenyi Biotec) using standard clinical parameters or an AutoMACS (Miltenyi Biotec) using the program posseld2.

Labelling and sorting of $CD34^+$ cells.

$CD34^+$ cells were resuspended in FACS buffer (5% FBS/1mM EDTA in DPBS)

containing 1/10 diluted FcBlock at a concentration of 10^7 cells/100 µL and stained on ice for 30 min with a cocktail of antibodies (CD34 subset markers – CD38, CD38, CD123, CD45RA, CD90: Lineage markers (LIN) – CD2, CD3, CD4, CD7, CD8a, CD10, CD11b, CD14, CD19, CD20, CD56, GlyA/GPA/CD235ab). Cells were sorted using a BD FACS ARIA II into the following populations: HSC-MPP (LIN^-, $CD34^+$, $CD38^{lo}$, $CD45RA^-$), CMP (LIN^-, $CD34^+$, $CD38^+$, $CD45RA^-$, $CD123^+$), GMP (LIN^-, $CD34^+$, $CD38^+$, $CD45RA^+$, $CD123^+$), MEP (LIN^-, $CD34^+$, $CD38^+$, $CD45RA^-$, $CD123^-$) (Figure S1A). Population gates were set using fluorescence minus one controls, and phenotypic purity checks were performed on collected cell fractions.

Functional validation of cell purity was carried out for a subset of experiments. Sorted cells were resuspended 1% methylcellulose supplemented with cytokines as described [2], then plated in triplicate at 500 cells per dish and incubated in a humidified 37°C incubator with 5% CO2 for 14 days. Three major types of colonies were counted: erythroid-lineage (BFU-E) colonies, myeloid-lineage (GM) colonies, and colonies with mixed-potential (GEMM) (Figure S1B).

Crosslinking and preparation of nuclei

Sorted cells were incubated in freshly prepared 1% formaldehyde in DPBS for 10 min at RT. Crosslinking was quenched by adding glycine to a final concentration of 0.125 M and incubating for 5 min at RT. Subsequent steps were performed at 4°C with cold buffers. Cells were washed then resuspended in cell lysis buffer (10mM Tris-Cl pH 8.0, 10mM NaCl, 0.2% Tergitol, supplemented with 1µg/mL leupeptin, 1mM Pefabloc SC, 10mM sodium butyrate) and incubated on ice for 10 minutes. Nuclei were centrifuged 1450g/10 min/4°C then snap frozen and stored at -80°C for later use.

Chromatin Immunoprecipitation (ChIP)

ChIP for H3K27ac and H3K4me3 was carried out essentially as described [3]. Nuclei (2–5 × 10^6/IP) were resuspended in 0.65mL nuclei lysis buffer (50mM Tris-Cl pH 8.0, 10mM EDTA, 1% SDS, protease inhibitors), incubated on ice for 10 minutes, with 0.4mL IP dilution buffer (20mM Tris-Cl pH 8.0, 2mM EDTA, 150mM NaCl, 1% Triton X-100, 0.01% SDS) and sonicated for 10 cycles in a Bioruptor Pico™ sonicator (Diagenode). Cleared supernatants were further diluted with 2.2mL IP dilution buffer, precleared with rabbit IgG then incubated overnight at 4^oC with 5–10 µg of antibody. Antibody-chromatin complexes were recovered using protein G-agarose beads (Roche). After washing, immunoprecipitated DNA was eluted from beads, crosslinks reversed, and DNA purified using phenol-chloroform-isoamyl alcohol. ChIP libraries were prepared by a commercial supplier (Novogene). Donor cells used in each experiment are listed in Table S1.

ChIPmentation

ChIPmentation (CM) was carried out as described [4] with minor modifications. Biological triplicate experiments were performed for TFs except where noted otherwise (Table S2). Five million nuclei were resuspended in 100µL sonication buffer (10mM Tris pH 8.0, 2mM EDTA, 0.25% SDS), sonicated for 10 cycles in a Bioruptor Pico™ sonicator (Diagenode) and diluted 1:1.5 in equilibrium buffer (10mM Tris pH 8.0, 233mM NaCl, 1.66% Triton X-100, 0.166% sodium deoxycholate, 1mM EDTA). Cleared supernatants were incubated overnight at 4^oC with 2µg of antibody, and antibody-chromatin complexes recovered using protein A/G magnetic beads. To improve signal to noise ratio for ERG CM only, we used a modified pull down protocol with two major differences: 1) nuclei lysates

were not cleared by centrifugation after sonication, and 2) anti-ERG antibody was pre-conjugated to magnetic protein A/G beads, and then incubated with nuclear lysate overnight at 4°C to recover antibody-chromatin complexes.

After extensive washing, bead-bound complexes were resuspended in tagmentation mixture (25µL reaction containing 1 µL of enzyme in 1X buffer, Illumina) and incubated at 37°C for 25minutes. Crosslinking was reversed and DNA purified using a MinElute PCR Purification Kit (Qiagen). Barcoding/adapter primers (Table S10) and KAPA™ HiFi HotStart Ready Mix (Roche) were used to amplify libraries; the number of PCR cycles used was empirically determined for each reaction. Amplified DNA was purified then size selected using AMPure XP beads (Beckman) and sequenced using a standard Illumina 2 x 150bp PE pipeline (Novogene).

HiC and HiChIP

Duplicate HiC and HiChIP libraries for each cell type were generated using the Arima Genomics HiC+ kit (Arima cat#A101020). Nuclei were lysed and chromatin digested with a restriction enzyme cocktail prior to end-filling with biotinylated nucleotides and ligation of proximal ends. For HiChIP, ligated fragments were then immunoprecipitated with the H3K27ac antibody. Biotinylated fragments were enriched and sheared prior to library preparation which was performed using Accel NGS 2S Plus DNA Library kit (Swift Biosciences).

Bioinformatic processing

Analyses were run using default parameters for each tool unless otherwise indicated. Bigwig files were visualized using the UCSC browser [5]. Reads were aligned to the GRCh38

genome accession GCF_000001405.26 (https://www.ncbi.nlm.nih.gov/assembly/GCF_000001405.26/). Heatmaps were plotted using seaborn unless otherwise indicated.

Processing ATAC sequencing data

SRA files were downloaded from GEO (GSE74912), converted to fastq format, and aligned to the GRCh38 genome. Mitochondrial and duplicate reads were removed (respectively) , reads shifted to account for Tn5-mediated excision, peaks called, and bigwig files generated (pipeline: SRA tools - BWA - samtools - picard MarkDuplicates - alignmentSieve - macs2 with a minimal threshold p-value of $1x10^{-5}$ - deeptools bamCoverage)[6,7]. To obtain a composite of all accessible regions in HSPCs, ATAC peaks from HSC-MPP, CMP, GMP, and MEP were merged using bedtools [8].

Processing ChIP sequencing data

ChIP fastq files were analysed as single end data. fastq files were processed to remove adapter sequences (cutadapt)[9], trimmed to 100 bp (Trim Galore), then aligned to GRCh38 (BWA). Reads were sorted (samtools) and duplicates removed (picard MarkDuplicates), then bam files from replicates merged (samtools merge). IgG bam files from the four cell types were merged to generate a single IgG control. Peaks were called (macs2 with a minimal threshold p-value of 1×10^{-5} and using the IgG track as the control), and RPKM-normalized bigwig files generated and plotted (deeptools bamCoverage, plotHeatmap) [10]. Composite plots showing ChIPseq signal at specific genomic regions were plotted in python as previously described [11].

Motif enrichment analysis

Motif enrichment analysis was performed using the FIMO tool from the MEME analysis suite [12] using ETS, GATA, RUNX, and E-Box motifs sourced from JASPAR [13] as a position weight matrix.

Analysis of combinatorial binding

Genomic locations with occupancy of multiple heptad TFs were identified by intersecting ChIP peak coordinates (bedtools intersect). To assess the significance of each combination we performed a bootstrapping analysis essentially as previously described [14]. Briefly, we applied a permutation test to address significance of combinatorial binding events between the seven transcription factors for all 119 possible binding patterns. We chose the merged ATAC peak set of 85,117 peaks to estimate the background distribution of combinatorial binding events. The standardized z-score metric was used to express the deviation of the combinatorial binding events in high confidence peaks from the expected mean (normalized by the standard deviation) of the background distribution.

Analysis of HiC and HiChIP data

HiChIP and HiC fastq files were processed and mapped to GRCh38, then PCR duplicates removed and contact matrices generated from the merged valid-pairs files (HiC-Pro hicpro2juicebox) [15]. Contact matrices (.hic files) were visualized using juicebox [16]. HOMER was used to identify compartments and TADs from balanced HiC-Pro contact matrices. The first principal component (PC1) was generated using runHiCpca.pl at 50 kb

bins. In addition, H3K4me3 and H3K27me3 bed files from the respective cell types were used to assign accurate compartment labelling. To further identify TADs, findTADsAndLoops.pl function was used to generate TAD calls for each replicate separately – and merge2Dbed.pl to generate a union of TADs identified in each replicate. HiChIP contact matrices were used to generate interaction pairs at 5 kb resolution (MaxHiC)[17] and the WashU browser was used for loop visualization [18]. Most interactions spanned distances >10 kb.

High confidence interactions (FDR ≤ 0.01) were used to generate a final list of promoter–regulator interactions. To map promoter-regulator interactions at heptad gene loci we identified HiChIP fragments which overlapped known promoters. Distal fragments that were linked to these promoters were intersected with ATAC peaks from the relevant populations to precisely map the contact region within 5kb HiCHIP fragments (Table S3). Contact regions were named according to their linear genomic distance upstream (-) or downstream (+) from the transcriptional start site (TSS).

Visualization of gene regulatory networks (GRNs)

GRNs were visualized with BioTapestry [19] using ChIPseq peak calls and HiChIP-derived promoter–regulatory links to construct the network maps.

Identifying differentially bound regions

Candidate regulatory elements (REs) were defined as regions displaying combinatorial binding of heptad factors with a positive z-score, indicating that the combination is observed at higher frequency than expected by random chance. DiffBind was used to identify regions showing a significantly higher (FDR ≤ 0.05) combinatorial TF signal in one cell type compared to all others [20]. Two criteria were used for linking differentially

bound REs with genes: the presence of a RE at a gene promoter, or within a HiChIP fragment that was in turn linked to a gene promoter. Gene lists derived using this method were subsequently used for GSEA, IPA, and single cell RNAseq analysis (Figure 4).

Analysis of bulk RNAseq data

Fastq files and count tables were downloaded from GEO (GSE75384) and fastq files aligned to GRCh38 (STAR) [21]. edgeR was used to normalise the count table and calculate log_2 CPM values [22] then derive a z-score of RNA expression.

Analysis of single cell RNAseq data

SCANPY [23] was used to process existing single-cell RNA sequencing data [24]. The SCANPY score_genes tool was used to generate a score for our gene sets, which was ultimately plotted on the original tSNE map generated by those authors.

TF occupancy at specific gene regulatory regions

Lists of genes associated with stem, myeloid, or erythroid function (Table S5B) were compiled from MSigDB genesets[25] (stem cell function – EPPERT_HSC_R[26], IVANOVA_HEMATOPOIESIS_STEM_CELL[27]: myeloid cell development - BIOCARTA_MONOCYTE_PATHWAY, BIOCARTA_GRANULOCYTES_PATHWAY, GOBP_GRANULOCYTE_DIFFERENTIATION, GOBP_GRANULOCYTE_MIGRATION, GOBP_GRANULOCYTE_ACTIVATION: erythroid cell development - HALLMARK_HEME_METABOLISM[28], GNATENKO_PLATELET_SIGNATURE[29],

BIOCARTA_PLATELETAPP_PATHWAY). Curated lists may not comprehensively catalogue every lineage specific gene. For this analysis promoter regions for each gene were defined as the ATAC peak occurring up to 10 kb upstream of the TSS. Distal fragments that were linked to these promoters in HiChIP datasets were intersected with ATAC peaks from the relevant population to define the distal regulatory element. Gene promoters lacking a looped distal region were excluded from this analysis. To determine the heptad TF signal for each distal region, we added together the average signal across each region from log_2-normalised bigwig tracks from each TF (bigWigAverageOverBed). If a looped distal regulator contained more than one ATAC peak, the TF signal from each peak region was averaged. We performed *k*-means clustering on the derived data using SciPy [30] and plotted the resulting heatmaps with seaborn.

<u>Clustering analysis</u>

The merged set of ATAC peaks which represent open chromatin regions across HSPCs were annotated using ChIP (heptad, PU.1, CTCF, ATAC, H3K27ac, H3K4me3, and H3K27me3) and ATAC signal from each individual cell type to create a dataframe of 85,100 rows and 52 columns, and the regions clustered with SCANPY using the Louvain method [23,31]. Gene associations for each ATAC region were predicted with the Genomic Regions of Enrichment Analysis Tool (GREAT) using the basal plus extension method with default parameters [32,33]. To identify regulatory regions preferentially used in specific cell types we compared TF signal at each region in HSC-MPP versus GMP, HSC-MPP versus MEP, and GMP versus MEP. We classified regions with log_2 fold change > 2 for each heptad factor in a cell type as cell-specific-regions. Regions identified by this method were subsequently used for machine learning models (Figure 7).

Machine learning analysis to predict cell type

We trained models using the R package XGBoost [34]. Briefly, we read a table of motif counts across the individual cell-specific-regions and took 70% of the peaks at random as the training set. We removed motifs with low variability and retained the remaining 30% of peaks as the test set. During the training, a series of decision trees were created such that a “loss function” was reduced (binary logistic in our case), to minimize cell type prediction error. Post training, prediction was performed on the test set. SHapley Additive exPlanation (SHAP) scores were calculated for every motif and peak used in the training set to indicate their respective contribution to the classification. A positive SHAP score for any given motif indicates that the presence of that motif in a region increases the probability that region belongs to the target cell type while a negative score indicates that the presence of that motif in a region increases the probability that region belongs to the background set (i.e., one of the other cell types). We then ranked motifs according to their importance by adding the absolute SHAP scores for every motif. To identify the direction of enrichment, we calculated the mean number of counts of every motif in the peaks that come from the target cell type or from the background peaks separately; if the mean was higher in the peaks from the target cell type those motifs were indicated as enriched in the target cell type and vice versa for the background set.

Analysis of γ retroviral integration site data

Late γ retroviral integration cluster genomic coordinates [1], representing viral integration sites (and thus potentially active regulatory elements) in long term repopulating human HSCs, were first overlapped with our merged ATAC peaks to generate genomic regions to use with our machine learning models (late γRV-IS, n = 2111). Of these, 395 (18.7%) corresponded to HSC-MPP-specific ATAC regions identified in our analysis; conversely 9.8% of our HSC-

MPP-specific sites corresponded to late γRV-IS. We next filtered out any regions that were part of the training sets for our models, leaving 1674 regions representing functionally validated regulatory regions that are active in hematopoietic stem cells with the capacity to engraft humans in a gene therapy setting (late γRV-IS). Late γRV-IS regions were scored against each machine learning model, and the predicted probabilities for each model plotted.

Data availability

A UCSC browser session for visualisation of chromatin occupancy and looping data is provided at http://genome.ucsc.edu/s/PimandaLab/Heptad_Regulome. We also provide a web tool for data exploration (http://unsw-data-analytics.shinyapps.io/CD34_Heptad_Regulome). Raw and processed sequencing files have been uploaded to GEO with accession # GSE231486.

References

1. Wunsche P, Eckert ESP, Holland-Letz T, et al. Mapping Active Gene-Regulatory Regions in Human Repopulating Long-Term HSCs. *Cell Stem Cell*. 2018;23(1):132-146 e139.

2. Schuller CE, Jankowski K, Mackenzie KL. Telomere length of cord blood-derived CD34(+) progenitors predicts erythroid proliferative potential. *Leukemia*. 2007;21(5):983-991.

3. Diffner E, Beck D, Gudgin E, et al. Activity of a heptad of transcription factors is associated with stem cell programs and clinical outcome in acute myeloid leukemia. *Blood*. 2013;121(12):2289-2300.

4. Schmidl C, Rendeiro AF, Sheffield NC, Bock C. ChIPmentation: fast, robust, low-input ChIP-seq for histones and transcription factors. *Nat Methods*. 2015;12(10):963-965.

5. Kent WJ, Sugnet CW, Furey TS, et al. The human genome browser at UCSC. *Genome Res*. 2002;12(6):996-1006.

6. Li H, Durbin R. Fast and accurate short read alignment with Burrows-Wheeler transform. *Bioinformatics*. 2009;25(14):1754-1760.

7. Li H, Handsaker B, Wysoker A, et al. The Sequence Alignment/Map format and SAMtools. *Bioinformatics*. 2009;25(16):2078-2079.

8. Quinlan AR, Hall IM. BEDTools: a flexible suite of utilities for comparing genomic features. *Bioinformatics*. 2010;26(6):841-842.

9. Martin M. Cutadapt removes adapter sequences from high-throughput sequencing reads. *EMBnetjournal*. 2011;17(1):10-12.

10. Ramirez F, Ryan DP, Gruning B, et al. deepTools2: a next generation web server for deep-sequencing data analysis. *Nucleic Acids Res*. 2016;44(W1):W160-165.

11. Thoms JAI, Truong P, Subramanian S, et al. Disruption of a GATA2-TAL1-ERG regulatory circuit promotes erythroid transition in healthy and leukemic stem cells. *Blood*. 2021;138(16):1441-1455.

12. Grant CE, Bailey TL, Noble WS. FIMO: scanning for occurrences of a given motif. *Bioinformatics*. 2011;27(7):1017-1018.

13. Castro-Mondragon JA, Riudavets-Puig R, Rauluseviciute I, et al. JASPAR 2022: the 9th release of the open-access database of transcription factor binding profiles. *Nucleic Acids Res*. 2022;50(D1):D165-D173.

14. Beck D, Thoms JA, Perera D, et al. Genome-wide analysis of transcriptional regulators in human HSPCs reveals a densely interconnected network of coding and noncoding genes. *Blood*. 2013;122(14):e12-22.

15. Servant N, Varoquaux N, Lajoie BR, et al. HiC-Pro: an optimized and flexible pipeline for Hi-C data processing. *Genome Biol*. 2015;16:259.

16. Durand NC, Shamim MS, Machol I, et al. Juicer Provides a One-Click System for Analyzing Loop-Resolution Hi-C Experiments. *Cell Syst*. 2016;3(1):95-98.

17. Alinejad-Rokny H, Ghavami Modegh R, Rabiee HR, et al. MaxHiC: A robust background correction model to identify biologically relevant chromatin interactions in Hi-C and capture Hi-C experiments. *PLoS Comput Biol*. 2022;18(6):e1010241.

18. Li D, Hsu S, Purushotham D, Sears RL, Wang T. WashU Epigenome Browser update 2019. *Nucleic Acids Res*. 2019;47(W1):W158-W165.

19. Longabaugh WJ. BioTapestry: a tool to visualize the dynamic properties of gene regulatory networks. *Methods Mol Biol*. 2012;786:359-394.

20. Stark R, Brown G. DiffBind: differential binding analysis of ChIP-Seq peak data

2011.

21. Dobin A, Davis CA, Schlesinger F, et al. STAR: ultrafast universal RNA-seq aligner. *Bioinformatics*. 2013;29(1):15-21.

22. Robinson MD, McCarthy DJ, Smyth GK. edgeR: a Bioconductor package for differential expression analysis of digital gene expression data. *Bioinformatics*. 2010;26(1):139-140.

23. Wolf FA, Angerer P, Theis FJ. SCANPY: large-scale single-cell gene expression data analysis. *Genome Biol*. 2018;19(1):15.

24. Setty M, Kiseliovas V, Levine J, Gayoso A, Mazutis L, Pe'er D. Characterization of cell fate probabilities in single-cell data with Palantir. *Nat Biotechnol*. 2019;37(4):451-460.

25. Liberzon A, Subramanian A, Pinchback R, Thorvaldsdottir H, Tamayo P, Mesirov JP. Molecular signatures database (MSigDB) 3.0. *Bioinformatics*. 2011;27(12):1739-1740.

26. Eppert K, Takenaka K, Lechman ER, et al. Stem cell gene expression programs influence clinical outcome in human leukemia. *Nat Med*. 2011;17(9):1086-1093.

27. Ivanova NB, Dimos JT, Schaniel C, Hackney JA, Moore KA, Lemischka IR. A stem cell molecular signature. *Science*. 2002;298(5593):601-604.

28. Liberzon A, Birger C, Thorvaldsdottir H, Ghandi M, Mesirov JP, Tamayo P. The Molecular Signatures Database (MSigDB) hallmark gene set collection. *Cell Syst*. 2015;1(6):417-425.

29. Gnatenko DV, Dunn JJ, McCorkle SR, Weissmann D, Perrotta PL, Bahou WF. Transcript profiling of human platelets using microarray and serial analysis of gene expression. *Blood*. 2003;101(6):2285-2293.

30. Virtanen P, Gommers R, Oliphant TE, et al. SciPy 1.0: fundamental algorithms for scientific computing in Python. *Nat Methods*. 2020;17(3):261-272.

31. Blondel VD, Guillaume J-L, Lambiotte R, Lefebvre E. Fast unfolding of communities in large networks. *Journal of Statistical Mechanics: Theory and Experiment*. 2008;2008(10):P10008.

32. McLean CY, Bristor D, Hiller M, et al. GREAT improves functional interpretation of cis-regulatory regions. *Nat Biotechnol*. 2010;28(5):495-501.

33. Tanigawa Y, Dyer ES, Bejerano G. WhichTF is functionally important in your open chromatin data? *PLoS Comput Biol*. 2022;18(8):e1010378.

34. Chen T, Guestrin C. XGBoost: A Scalable Tree Boosting System. Proceedings of the 22nd ACM SIGKDD International Conference on Knowledge Discovery and Data Mining. San Francisco, California, USA; 2016.
